# Serpin-1a and serpin-6 regulate the Toll pathway immune homeostasis by synergistically inhibiting the Spätzle-processing enzyme CLIP2 in silkworm, *Bombyx mori*

**DOI:** 10.1371/journal.ppat.1011740

**Published:** 2023-10-18

**Authors:** Huawei Liu, Jiahui Xu, Luoling Wang, Pengchao Guo, Zhangchen Tang, Xiaotong Sun, Xin Tang, Wei Wang, Lingyan Wang, Yang Cao, Qingyou Xia, Ping Zhao

**Affiliations:** 1 Integrative Science Center of Germplasm Creation in Western China (CHONGQING) Science City, Biological Science Research Center, Southwest University, Chongqing, China; 2 Key Laboratory for Germplasm Creation in Upper Reaches of the Yangtze River, Ministry of Agriculture and Rural Affairs, Chongqing, China; 3 Chongqing Key Laboratory of Chinese Medicine & Health Science, Chongqing Academy of Chinese Materia Medica, Chongqing College of Traditional Chinese Medicine, Chongqing, China; University of Cambridge, UNITED KINGDOM

## Abstract

The Toll receptor signaling pathway is an important innate immune response of insects to pathogen infection; its extracellular signal transduction involves serine protease cascade activation. However, excessive or constitutive activation of the Toll pathway can be detrimental. Hence, the balance between activation and inhibition of the extracellular protease cascade must be tightly regulated to achieve favorable outcomes. Previous studies have shown that serpins—serine protease inhibitors—negatively regulate insect innate immunity by inhibiting extracellular protease cascade signaling. Although the roles of serpins in insect innate immunity are well described, the physiological mechanisms underlying their synergistic effects remain poorly understand. Here, we characterize the molecular mechanism by which serpin-1a and serpin-6 synergistically maintain immune homeostasis of the silkworm Toll pathway under physiological and pathological conditions. Through *in vitro* biochemical assays and *in vivo* bioassays, we demonstrate that clip-domain serine protease 2 (CLIP2), as the Toll cascade-activating terminal protease, is responsible for processing proSpätzle1 to induce the expression of antimicrobial peptides. Further biochemical and genetic analyses indicate that constitutively expressed serpin-1a and inducible serpin-6 synergistically target CLIP2 to maintain homeostasis of the silkworm Toll pathway under physiological and pathological conditions. Taken together, this study provides new insights into the precise regulation of Toll cascade activation signals in insect innate immune responses and highlights the importance and complexity of insect immune homeostasis regulation.

## Author summary

Like other invertebrates, insects rely on their innate immune system to respond effectively to immune challenge. The insect Toll signaling pathway mediates resistance to bacterial infections by regulating the expression of antimicrobial peptides (AMPs). The Toll pathway-activating signals transduced by the serine protease cascade are regulated by serpin. In this study, we show that CLIP2 functions as an extracellular terminal protease of the domestic silkworm Toll pathway, stimulating the expression of AMPs by processing proSpätzle1 and enhancing the antibacterial activity of hemolymph. Our results indicate that serpin-1a and serpin-6 can form covalent complexes with CLIP2 to inhibit proSpätzle1 cleavage, thereby reducing the expression of gloverin2. Serpin-1a and serpin-6 synergistically target CLIP2 in silkworm hemolymph under physiological or pathological conditions. Moreover, knocking out *serpin-1* increases CLIP2 abundance and background expression levels of AMPs in the absence of immune challenge. Meanwhile, immune stimulation after *serpin-1* knockout causes compensatory serpin-6 expression, which in turn leads to the expression level of AMPs and resistance to pathogens being close to that of wild-type individuals. In summary, our results reveal the synergistic regulatory mechanism of serpin-1a and serpin-6 in maintaining the immune homeostasis of the Toll pathway in silkworms.

## Introduction

Insects are a major group of arthropods, and the most diverse, numerous, and widely distributed group of animals on Earth. During their evolutionary process, insects have evolved a complex and efficient innate immune system to resist pathogen invasion [1–3]. Their immune system comprises interconnected cellular and humoral responses, including phagocytosis, encapsulation, activation of proteolytic cascades, activation of prophenoloxidase (proPO), and synthesis of potent antimicrobial peptides (AMPs) [4–6]. In particular, humoral immunity regulates the synthesis of AMPs through the Toll and IMD signaling pathways and induces melanization reactions via the proPO system [1,7]. Extracellular serine protease (SP) cascades are key components of the humoral immune signal transmission process, which mediates rapid defense responses to pathogens by activating the Toll pathway and the proPO system in insects [8–11].

The immune extracellular SP cascades of Toll pathway are best studied in *Drosophila melanogaster*, *Tenebrio molitor*, and *Manduca sexta* [8,9,12]. In *D*. *melanogaster*, genetic and RNA interference analyses have revealed that an SP cascade comprising modular SP (ModSP, MSP), Grass, and SPE (Spätzle-processing enzyme) participates regulating the Toll pathway [13,14]. In *T*. *molitor*, *in vitro* biochemical studies have demonstrated that the MSP-SAE (SPE-activating enzyme)-SPE SP cascade mediates activation of the Spätzle (Spz)-Toll pathway [10]. Moreover, detailed biochemical studies have confirmed that *M*. *sexta* SP cascade of hemolymph protease 14 (HP14)-HP21-HP5-HP6-HP8 leading to the activation of Spz-Toll pathway [11,15,16]. Among the SPs involved in the hydrolysis cascade, excluding immune pathway-initiating SPs (MSP, and HP14) that receive upstream signals, other SPs are clip-domain SPs (CLIPs) that contain one or more amino-terminal clip domains [8]. Terminal CLIPs of extracellular signaling cascades are responsible for processing the proSpz protein to produce Toll ligands during the immune response. However, in other insects, knowledge regarding which CLIPs mediate Spz-Toll pathway activation is lacking.

The Toll pathway is mediated by extracellular SP cascades, which are negatively regulated by serpins [8,11,17–20]. Serpins are a superfamily of proteins, typically 350–500 amino acids in length with a molecular weight of 40–50 kDa, that generally function as suicide inhibitors by forming covalent complexes with target proteases [17]. In *D*. *melanogaster*, the serpin-43Ac down-regulates fungal virulence factor-induced Toll signaling by inhibiting the protease Persphone, whereas serpin-1 regulates the gram-negative bacteria binding protein 3 (GNBP3)-dependent Toll signaling pathway by inhibiting the SP Grass [21,22]. In *T*. *molitor*, serpin-40, serpin-55, and serpin-48 form three pairs of protease-serpin complexes with MSP, SAE, and SPE, respectively, to cooperatively regulate the Toll-signaling pathway [19]. In addition, *T*. *molitor* serpin-93, which contains two tandemly arrayed serpin domains, regulates all SPs in the extracellular cascade of the Toll pathway by forming a complex with SPE through its N-terminal serpin domain and a complex with MSP and SAE via the C-terminal serpin domain [23]. In *M*. *sexta*, the immune extracellular SP cascades of the Toll pathway (HP14-HP21-HP5-HP6-HP8) are regulated by several serpins. *M*. *sexta* serpin-4, serpin-1A, and serpin-1J jointly regulate HP5 activity, serpin-1J and serpin-6 jointly regulate HP8 activity, while serpin-12, serpin-4, and serpin-5 form covalent complexes with HP14, HP21, and HP6, respectively [11,18,24,25]. Although the extracellular signals transduced by the SP cascade are known to be regulated by serpins, with each SP being negatively regulated by one or more serpins, there is a lack of systematic research on how these serpins synergistically regulate target proteases under physiological and pathological conditions to maintain insect immune homeostasis.

Owing to the large size, ease of culture and well-established genetic manipulation methods, the domesticated silkworm, *Bombyx mori*, is a common model organism for lepidopterans [26–29]. Although 34 serpins and 26 CLIPs have been identified in silkworms, few functions have been clarified [30–32]. Serpin-5 functions as a negative regulator of proPO and AMP-producing pathways by forming covalent complexes with silkworm HP6 (CLIP25) and SP21 (CLIP20) [33]. Similarly, *B*. *mori* serpin-6 and serpin-15 are involved in the negative regulation of proPO activation and AMP expression [34,35]. Meanwhile, serpin-28 knockdown impacts AMP gene expression, whereas serpin-32 inhibits the spontaneous melanization by blocking proPO activation in silkworms [36,37]. Owing to the incomplete establishment of extracellular SP cascades of humoral immunity, although the functions of several serpins in the silkworm have been identified, the physiological target proteases of all serpins, excluding serpin-5, are unknown.

CLIP11 (BmSPH-1) plays a dual role in the melanization of the pupal cuticle and the immune response of hemocytes [38], whereas CLIP13 (BmSP95) is inducible by 20E and is involved in silkworm cuticle remodeling during molting and metamorphosis [39,40]. Moreover, *B*. *mori* CLIP1 (PPAE, BmSP133) is involved in the activation of proPO [41,42]. Meanwhile, CLIP2 (BAEEase, BmSP127)—the *Bombyx* homolog of *Drosophila* SPE—is a candidate proSpz1 activator that is activated by upstream SP cascade components in the presence of peptidoglycan (PGN) and β-1-3-glucan in silkworm hemolymph [43,44]. However, it remains unclear whether CLIP2 cleaves proSpz1 and participates in silkworm humoral immunity.

Here, we used the model insect silkworm to explore the synergistic regulatory mechanism between serpins in immune homeostasis maintenance. We first characterized the immune function of CLIP2 in silkworm Toll pathway, and then identified and validated its physiological regulatory factors serpin-1a and serpin-6. Furthermore, we systematically investigated how serpin-1a and serpin-6 synergistically regulate CLIP2 to maintain the homeostasis of the Toll pathway under normal and infection conditions.

## Results

### Expression patterns of CLIP2, Spätzle1 (Spz1) and gloverin2 in silkworm fat body and hemolymph

**Fig 1:**
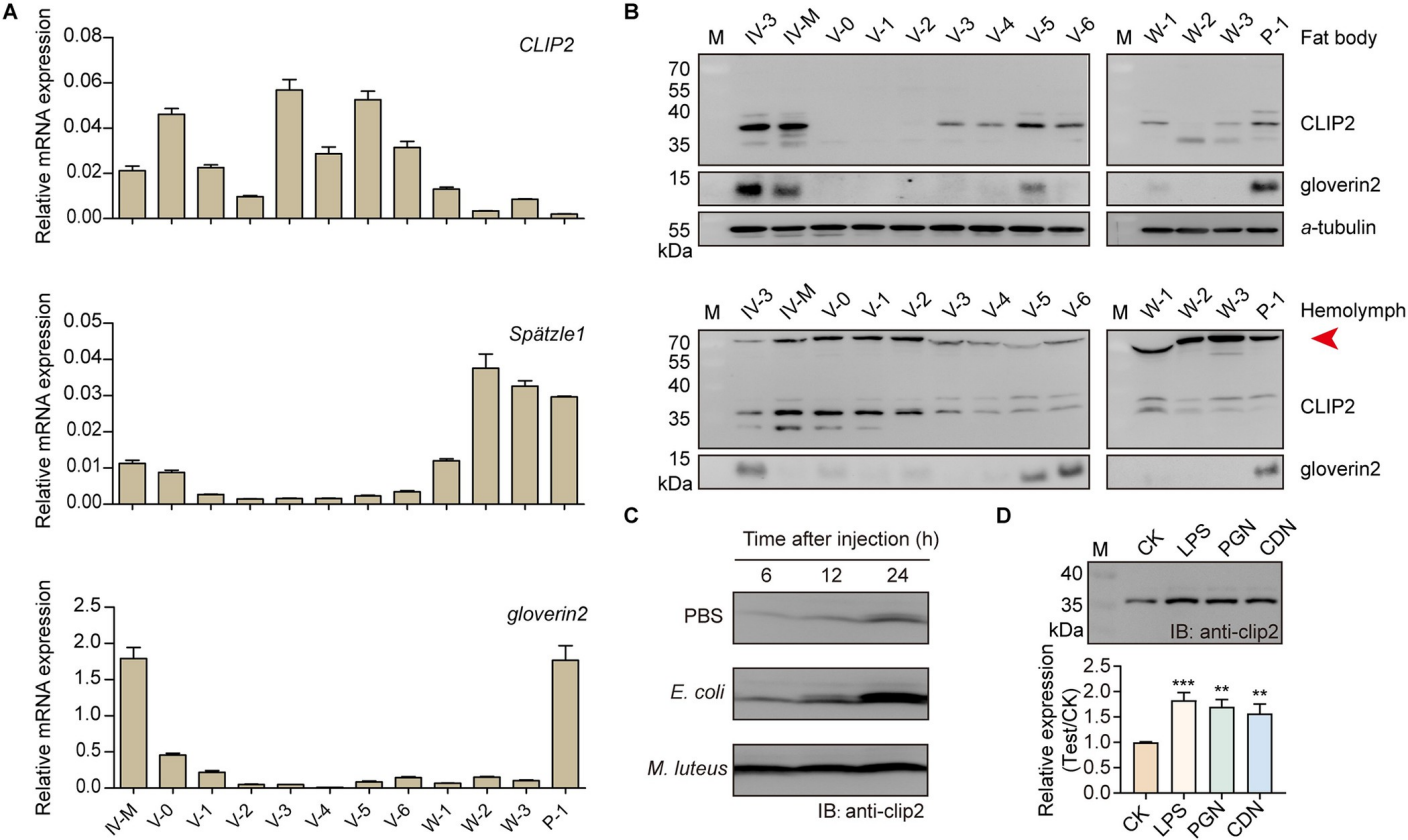
Expression patterns of immune-related molecules CLIP2, Spätzle1, and gloverin2 in silkworm immune tissues. Expression analysis of immune-related molecules in the fat body and hemolymph of silkworm at different developmental stages (A and B). *a*-tubulin was used as the reference protein. IV-3: day 3 of fourth-instar larvae; IV-M: molting of fourth-instar larvae; V-0~V-6: days 0–6 of fifth-instar larvae; W-1–W-3: days 1–3 after wandering; P-1: day 1 after pupation. The lower bands recognized by CLIP2 antibodies in fat body and hemolymph may be degraded bands resulting from CLIP2 being unspecifically cleaved. The red arrow indicates a covalent complex formed by CLIP2 and its specific inhibitor. (C) Effects of *Escherichia coli* and *Micrococcus luteus* on the expression of CLIP2 in silkworm hemolymph. (D) Effects of bacteria, lipopolysaccharide (LPS), peptidoglycan (PGN) and curdlan (CDN) on the expression of CLIP2 in silkworm hemolymph. Error bars represent mean ± SD (n = 3). **P < 0.01; ***P < 0.001. M: protein molecular weight marker.

To analyze the activation level of the humoral Toll pathway in silkworms during normal development, the expression of the immune-related molecules CLIP2, Spz1 and gloverin2 was quantified in the fat body and hemolymph by real-time quantitative PCR (RT-qPCR) and immunoblotting. RT-qPCR results showed that *CLIP2* mRNA was highly expressed in the fat body at the larval stage, while *Spz1* and *gloverin2* mRNA were highly expressed in the fat body during the fourth-instar molting stage, newly molted fifth-instar stage, and wandering stage (Fig 1A). Immunoblot results further revealed that CLIP2 protein was continuously detected in the fat body and hemolymph at different developmental stages, while gloverin2 protein was only detected at the end of the fourth and fifth-instar stages, and on day 1 of the pupal stage (Fig 1B). A lagging band with a molecular weight > 70 kDa was also detected in the hemolymph at different developmental stages using the CLIP2 antibody (Fig 1B). Based on the regulatory characteristics of CLIP activity [17], it is speculated that this band is a covalent complex formed by CLIP2 and its specific inhibitor. These results indicate that the cascade molecule CLIP2 has a high level of background expression in silkworm hemolymph, and its activity may be continuously regulated.

Furthermore, *Escherichia coli*, *Micrococcus luteus* and pathogenic molecular patterns (PAMPs; lipopolysaccharide, LPS; peptidoglycan, PGN; and curdlan, CDN) were injected into the hemocoel of day 3 of the fifth-instar larvae to analyze the response of CLIP2 to immune stimulation. Immunoblotting results showed that CLIP2 protein levels were significantly increased in the hemolymph induced by pathogens or PAMPs, and the induction effect of *M*. *luteus* or LPS was more pronounced (Fig 1C and 1D). These results showed that CLIP2 not only persisted in the silkworm hemolymph, but its expression level and form were also significantly affected by pathogen invasion. It is suggested that the expression patterns of CLIP2 may enable it to respond rapidly to immune stimulation and increase the level of silkworm humoral immunity.

### Cleavage of silkworm proSpätzle1 (proSpz1) by CLIP2

ProSpz is activated by DmSPE and MsHP8 in *D*. *melanogaster* and *M*. *sexta*, respectively [24,44]. Our previous evolutionary analysis showed that silkworm CLIP2 clusters in the same clade as DmSPE and MsHP8 [30]. Based on sequence similarity and activation characteristics, Jang *et al*. speculated that silkworm BAEEase (CLIP2) is a candidate proSpz1 activator [44]. To test this hypothesis, we assessed proSpz1 cleavage by CLIP2 *in vitro*.

**Fig 2:**
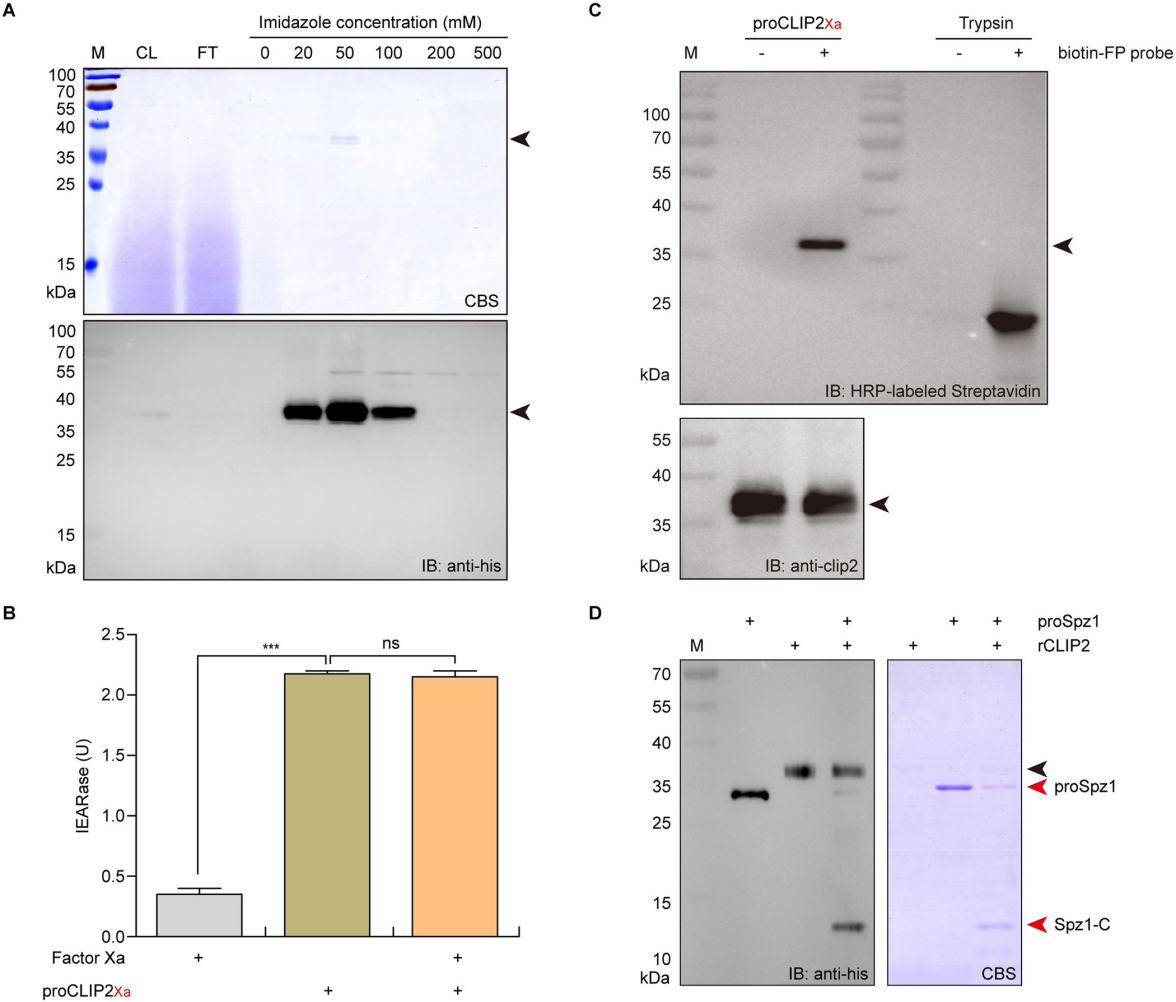
*In vitro* analysis of proSpätzle1 (proSpz1) cleavage by CLIP2. (A) Purification of recombinant proCLIP2_Xa_ protein by Ni-NTA (nitrilotriacetic acid) affinity chromatography with a stepwise imidazole gradient. CL: crude extract; FT: flow-through; 20–500: elution fractions of a stepwise imidazole gradient. The arrow indicates the recombinant proCLIP2Xa. M: protein molecular weight marker; CBS: commassie blue staining; IB: immunoblotting. The hydrolysis activity of recombinant proCLIP2_Xa_ was detected using polypeptide substrate Ile-Glu-Ala-Arg-p-nitroanilide (IEARpNA) (B) and the activity-based probe desthiobiotin-FP (C). ProCLIP2_Xa_ (0.75 μg) was pre-incubated with factor Xa (1 μg). Subsequently, the catalytic activity of factor Xa (1 μg), proCLIP2_Xa_ (0.75 μg), and pre-incubation solution of both were detected by a multifunctional microplate reader using IEARpNA as a substrate. Desthiobiotin-FP probe can specifically covalently label the active serine site of serine hydrolase. (D) Proteolytic activation of proSpz1 by recombinant CLIP2 (rCLIP2). The black arrow indicates the rCLIP2, the red arrow indicates the proSpz1 and the C-terminal fragment of proSpz1 (Spz1-C). Error bars represent mean ± SD (n = 3). ***P < 0.001.

Recombinant proCLIP2_Xa_ secreted by GS115 yeast cells was purified using Ni-nitrilotriacetic acid (Ni-NTA) affinity chromatography. Sodium dodecyl sulfate-polyacrylamide gel electrophoresis (SDS-PAGE) and immunoblot analyses showed that proCLIP2_Xa_ was primarily eluted in fractions containing 20, 50, and 100 mM imidazole; and the molecular weight of the purified proCLIP2_Xa_ was approximately 37 kDa (Fig 2A), which was similar to that of the active form of *M*. *sexta* HP8 [15,16].

We speculate that purified proCLIP2_Xa_ is activated after its secretion into the yeast culture medium. To verify this hypothesis, the hydrolytic activity of purified proCLIP2_Xa_ was determined using the polypeptide substrate Ile-Glu-Ala-Arg-*p*-nitroanilide (IEAR*p*NA) and the activity-based probe desthiobiotin-FP (Thermo Scientific, USA). Incubation of purified proCLIP2_Xa_ with factor Xa did not result in the appearance of new protein bands (S1B Fig). Meanwhile, the enzyme activity assay further showed that purified proCLIP2_Xa_ exhibited IEAR enzyme activity, which was not significantly impacted following incubation with factor Xa (Fig 2B).

Given that the FP probe has a reactive fluorophosphate group that can specifically and covalently label the active serine site of serine hydrolase [45], an activity-based probe labelling assay was performed. Similar to commercial trypsin, purified proCLIP2_Xa_ was labeled with desthiobiotin-FP, indicating that it had serine hydrolase activity (Fig 2C). These results indicate that purified proCLIP2_Xa_ is an active form cleaved by unknown factors in yeast culture medium. For clarity, recombinant CLIP2 (rCLIP2) was used to represent the purified proCLIP2_Xa_ protein in subsequent experiments.

Subsequently, the recombinant proSpz1 protein (His-tag added to the carboxyl-terminus) was purified from the inclusion bodies using Ni-NTA affinity chromatography. According to our previous study [46], the purified proSpz1 inclusion body protein was refolded using gradient dialysis to obtain the soluble proSpz1 protein (S2 Fig). Finally, rCLIP2 was mixed with proSpz1 to analyze proSpz1cleavage. SDS-PAGE and immunoblot results showed that after incubation with rCLIP2, the 34 kDa proSpz1 band disappeared and a 13 kDa C-terminal (Spz1-C) fragment, similar in size to the product of proSpz-1A cleaved by *M*. *sexta* HP8 [16], was produced (Fig 2D). These results indicate that CLIP2 is an activating protease of *B*. *mori* proSpz1.

### CLIP2 cleavage proSpz1 enhances the expression of antibacterial peptides and the antibacterial activity of hemolymph

Considering that our results suggested that CLIP2 may be involved in the activation of the Toll signaling pathway via activation of *B*. *mori* proSpz1. We investigated whether CLIP2 induces the expression of antimicrobial peptide genes in *B*. *mori*. To this end, we performed two groups of injection experiments with recombinant proteins.

**Fig 3:**
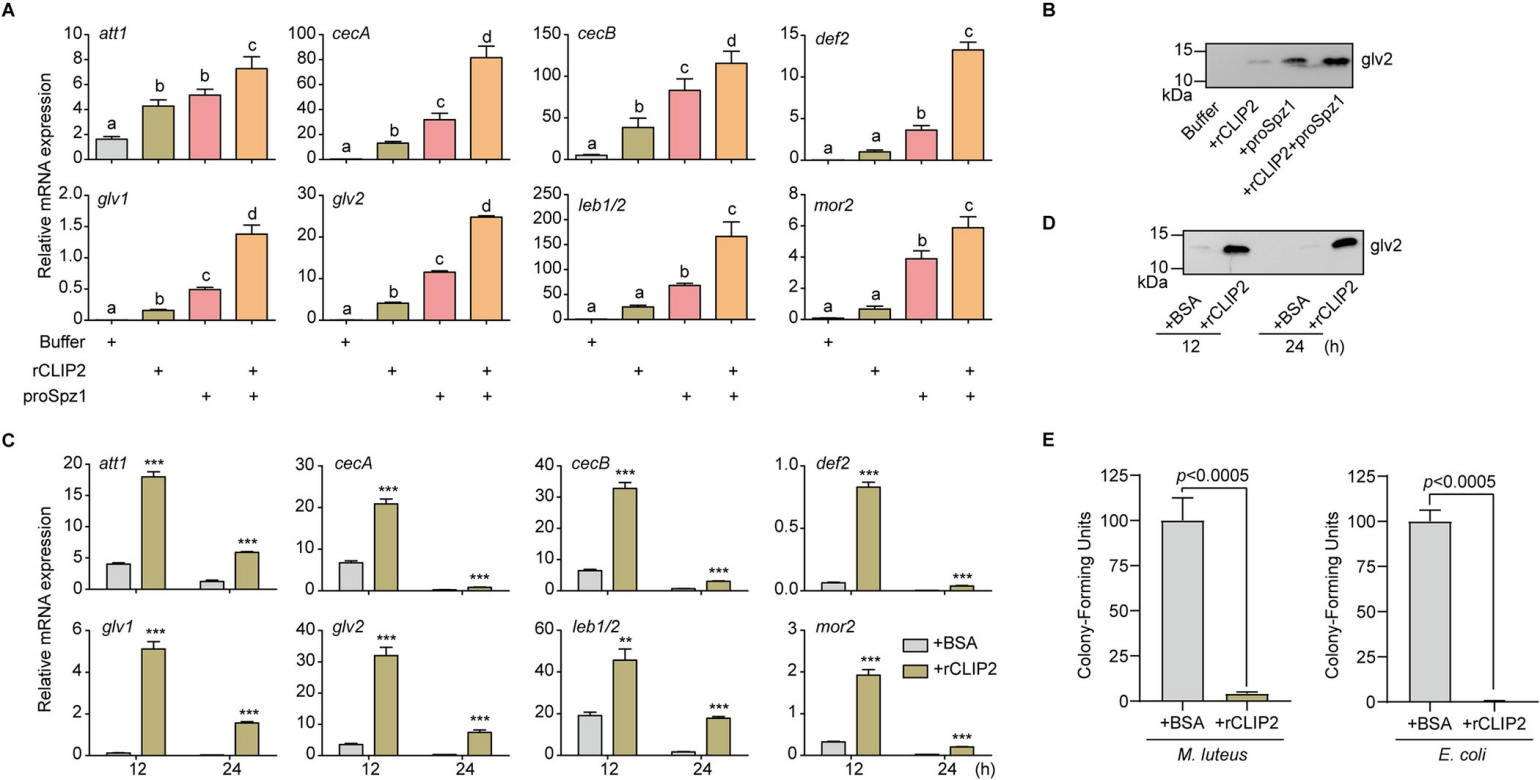
Effects of proSpätzle1 (proSpz1) and CLIP2 injection on the humoral immune response. Day 2 of fifth-instar larvae were injected with buffer, CLIP2, proSpz1, the incubation mixture of CLIP2 and proSpz1. At 12 h after injection, the fat body and hemolymph of each group were collected. The expression levels of antibacterial peptides (AMPs) were determined by RT-qPCR (A) and immunoblotting (B). Error bars show mean ± SD (n = 3). Different letters represent the significant differences (one-way ANOVA followed by Tukey’s test, *P* < 0.05). Furthermore, larvae at day 1 of the wandering stage were injected with CLIP2 or BSA. At 12 h and 24 h after injection, the fat body and hemolymph of each group were collected. The expression levels of antibacterial peptides (AMPs) were determined by RT-qPCR (C) and immunoblotting (D). Size and positions of molecular mass standards are indicated to the *left* of each blot. (E) Hemolymph collected 12 h post-injection of CLIP2 was used to determine the antimicrobial activity against *Micrococcus luteus* (*left*) and *Escherichia coli* (*right*). Error bars represent mean ± SD (n = 3). *** *P* < 0.001.

At the fifth-instar larval stage, with low expression levels of *Spz1* in the fat body, rCLIP2, proSpz1, an incubation mixture of rCLIP2 and proSpz1, or phosphate buffered saline (PBS) was injected into the larvae’s coelomic cavity. After 12 h, RNA was isolated from the fat body to measure AMP expression, and hemolymph was collected to quantify protein levels. RT-qPCR results showed that the transcript levels of *attacin1*, *cecropinA*, *cecropinB*, *defensin2*, *gloverin1*, *gloverin2*, *lebocin1/2*, and *moricin2* were significantly increased following injection of the rCLIP2 and proSpz1 mixture compared with buffer, rCLIP2, or proSpz1 (Fig 3A). Transcript levels of *attacin1*, *cecropinA*, *cecropinB*, *gloverin1*, and *gloverin2* were also significantly increased after the injection of rCLIP2 or proSpz1 compared with buffer (Fig 3A), which may be due to the interaction between the recombinant proteins and endogenous proSpz1 or CLIP2. Immunoblot results showed that compared with the injected buffer, gloverin2 protein levels significantly increased in turn in the hemolymph injected with rCLIP2, rproSpz1, and their both, which was consistent with the gloverin2 transcript level data (Fig 3B). These results indicate that the products of proSpz1 proteolytic cleavage by CLIP2 act as cytokines that activate the Toll pathway and promote AMP expression in silkworms.

At the wandering stage, with high expression levels of *Spz1* in the fat body, bovine serum albumin (BSA) or rCLIP2 was injected into the silkworm coelomic cavity. After 12 and 24 h, RNA was isolated from the fat body to measure the expression levels of AMPs, and the hemolymph was collected to quantify the protein levels and antimicrobial activity. RT-qPCR results showed that the transcript levels of *attacin1*, *cecropinA*, *cecropinB*, *defensin2*, *gloverin1*, *gloverin2*, *lebocin1/2*, and *moricin2* significantly increased after the injection of rCLIP2 as compared with the injection of BSA, and the increased levels were higher 12 h after injection (Fig 3C). Immunoblot results further revealed that, compared with BSA, the hemolymph injected with rCLIP2 contained significantly higher levels of gloverin2 protein (Fig 3D). Further antibacterial activity assays showed that the injection of rCLIP2 significantly enhanced the antibacterial activity of the hemolymph against *M*. *luteus* and *E*. *coli* (*P* < 0.0005) (Fig 3E). These results indicate that CLIP2 proteolytically cleave proSpz1, promote the expression of AMPs in the fat body, and enhance the antibacterial activity of the hemolymph.

### Serpin-1a and serpin-6 cooperatively inhibit proSpz1 cleavage by CLIP2

**Fig 4:**
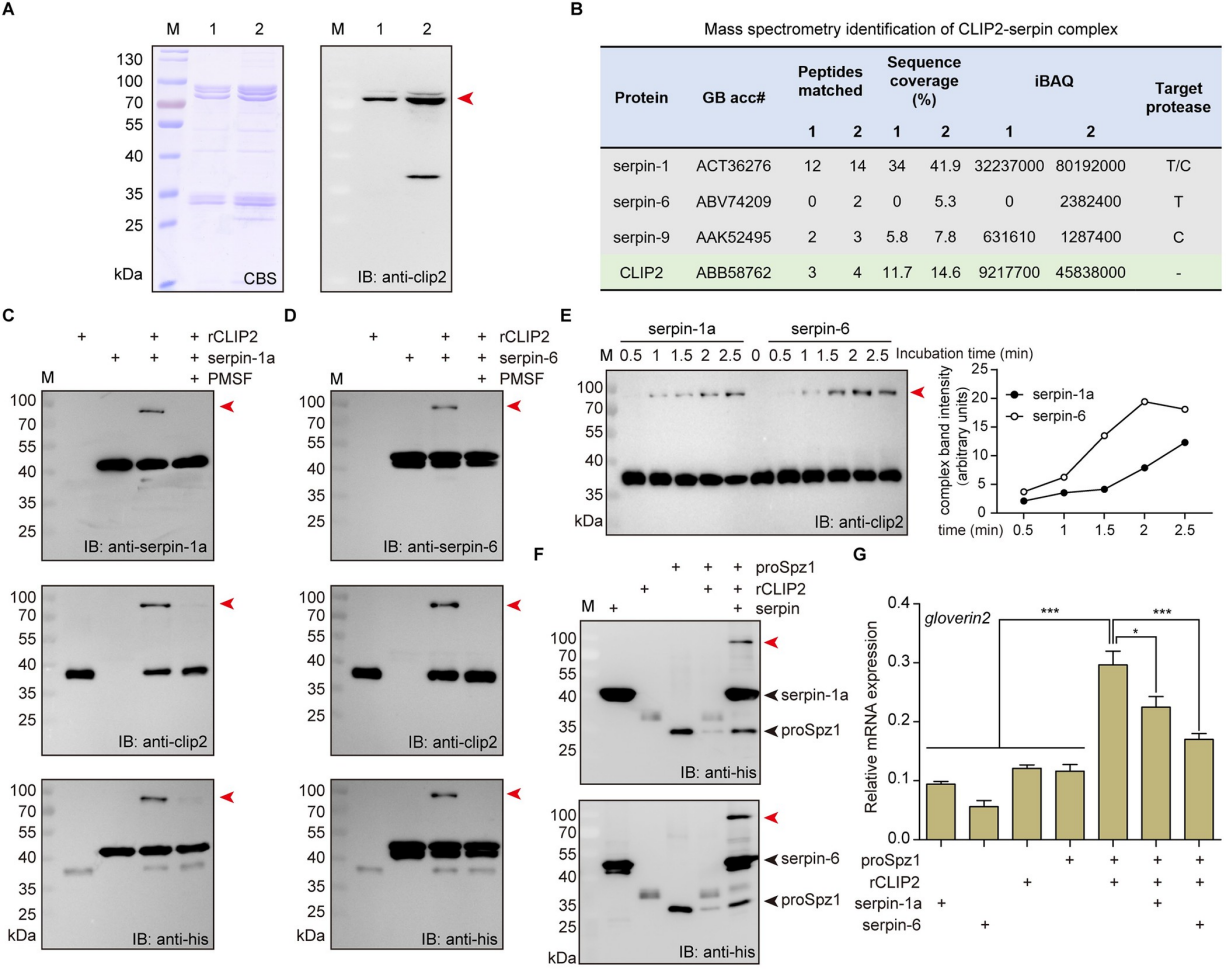
Identification and inhibitory activity analysis of physiological regulators of CLIP2. (A) Immunoprecipitated purified CLIP2-inhibitor complexes were subjected to SDS-PAGE and were detected by coomassie blue staining (CBS) or immunoblotting (IB). CLIP2 and associated proteins isolated from normal hemolymph (lane 1) or *Micrococcus luteus*-treated hemolymph (lane 2). The red arrow indicates the potential CLIP2-serpin complex. Coomassie blue stained bands corresponding to potential complexes were excised for trypsin digestion followed LC-MS/MS analysis; the identified CLIP and serpins were listed in (B). iBAQ (intensity-based absolute quantification) intensity was used to estimate the abundance of each protein. The target protease type of serpin is based on previous studies [31], with T for trypsin and C for chymotrypsin. SDS-stable complex formation between rCLIP2 and serpin-1a (C) or serpin-6 (D). rCLIP2 (200 ng) was incubated with corresponding serpins at room temperature for 5 min under a molar mass ratio of 1:5 (rCLIP2: serpins). The samples were subjected to SDS-PAGE and immunoblot analysis using antibodies against serpin-1a (*upper*), serpin-6(*upper*), CLIP2 (*middle*), and His-tag (*lower*). Red arrows indicate the CLIP2-serpin complex. (E) rCLIP2 (250 ng) was incubated with serpin-1a or serpin-6 at room temperature for 0, 0.5, 1, 1.5, 2, and 2.5 min under a molar mass ratio of 1:5 (rCLIP2: serpins). The reaction mixtures were separated by SDS-PAGE, followed by immunoblot analysis with antibodies against CLIP2. The red arrow indicates the CLIP2-serpin complex. Intensity of the ~80 kDa band corresponding to CLIP2-serpin complex was analyzed using ImageJ, and plotted against incubation time. (F) Serpin-1a (*upper*) or serpin-6 (*lower*) inhibits the cleavage of proSpz1 by CLIP2. CLIP2 (100 ng) was incubated at room temperature for 5 min with a 5-fold molar excess of serpin-1a or serpin-6, then incubated with proSpz1 (1 μg) at room temperature for 30 min. The samples were subjected to SDS-PAGE and immunoblotting using anti-His-tag antibodies. Red arrows indicate the CLIP2-serpin complex. Black arrows indicate the proSpz1 or serpin. In addition, the samples from each of the above groups were added to the BmN cell culture medium; the cultured BmN cells were collected 24 h later to detect the expression of the antimicrobial peptide gene gloverin2 by RT-qPCR (G). Error bars represent mean ± SD (n = 3). *P < 0.05; *** *P* < 0.001.

In the extracellular regulation of insect humoral immunity, extracellular cascade proteases are typically regulated by specific serpins that form covalent complexes with target proteases [17]. Based on this feature, we performed co-immunoprecipitation using a polypeptide antibody against CLIP2 to identify its regulatory factors in the silkworm hemolymph. Immunoblotting results showed that CLIP2 antibodies captured more covalent complexes of CLIP2 with its inhibitors in the hemolymph induced by *M*. *luteus* than in non-induced hemolymph (Fig 4A). The expression pattern and immunoprecipitation results suggested that the activity of CLIP2 was continuously regulated in the absence an immune challenge and was more strictly regulated after pathogen infection (Figs 1B and 4A).

To identify the regulatory factors of CLIP2, we performed LC-MS/MS analysis of SDS-PAGE gel strips corresponding to the location of the immunoblotting complex. The identified proteins and their intensities are listed in S1 Table. After analyzing the identified protein data, we identified serpin-1, serpin-6, serpin-9, and CLIP2 from the complex bands, and serpin-6 was only identified in the complex of induced hemolymph (Fig 4B). Previous studies have found that CLIP2 can hydrolyze the trypsin-prototypic substrate BAEE with a substrate-specific pocket composed of residues D311, G340, and G351, indicating that it is trypsin-type SP [30,44]. The P1-P1’ sites in the reactive center loop (RCL) region of the silkworm serpin protein showed that serpin-6 may inhibit trypsin-type SPs, serpin-9 may inhibit chymotrypsin-like SPs, and among the four serpin-1 variants (serpin-1a–d), only serpin-1a may inhibit trypsin-type SPs [31]. Therefore, serpin-1a and serpin-6 may participate in the regulation of CLIP2 activity in silkworm hemolymph.

To further investigate the interactions of serpin-1a and serpin-6 with CLIP2, we purified recombinant serpin-1a and serpin-6 proteins (S3A and S3B Fig). The inhibitory activity assay showed that serpin-1a significantly inhibited the activities of trypsin and papain, whereas serpin-6 significantly inhibited the activities of trypsin and chymotrypsin (S3C and S3D Fig). For serpin-1a, site-directed mutagenesis and activity assays showed the E329A and R340A mutants had fully lost its inhibitory activity against trypsin compared to that of the wild type and S341A (S3E Fig). Furthermore, serpin-1a or serpin-6 incubated with rCLIP2 formed higher molecular weight complexes as identified by immunoblotting (Fig 4C and 4D). The interaction of serpin-1a and rCLIP2 resulted in the formation of a ~80 kDa complex band. The combined apparent molecular mass of recombinant serpin-1a (42.6 kDa) and rCLIP2 (37 kDa) was similar to that of the complex (~80 kDa). This complex band was recognized by antibodies against serpin-1a, CLIP2 and His-tag (Fig 4C), indicating that it was composed of these two proteins. However, the R340A mutant of serpin-1a cannot form a covalent complex with rCLIP2 (S3F Fig). This indicates that R340 is the P1 site targeting trypsin in serpin-1a RCL. In addition, a complex band at ∼80 kDa was also observed between the recombinant serpin-6 (47.3 kDa) and rCLIP2 (37 kDa), which was recognized by antibodies against serpin-6, CLIP2 and His-tag (Fig 4D). These results confirm that serpin-1a and serpin-6 can form an SDS-stable complex with active CLIP2 and indicate that serpin-1a and serpin-6 are regulators of CLIP2 activity.

To examine the rate of complex formation between rCLIP2 and serpin-1a or serpin-6, we mixed serpin-1a or -6 with rCLIP2 at a molar ratio of 5:1, and stopped the reaction at different times. The immunoblot results showed that a pronounced complex could be detected by 1 min after mixing the serpin and rCLIP2, and the band gradually increased in intensity with incubation time extended (Fig 4E). Compared with serpin-1a, the rate of formation of the complex between serpin-6 and rCLIP2 is faster within 2.5 minutes, and the accumulation is also greater. These results indicate serpin-6 interacted with rCLIP2 faster and more effectively than serpin-1a.

Given that CLIP2 proteolytically cleaves proSpz1 and promotes AMP expression (Figs 2 and 3), we analyzed the effects of serpin-1a and serpin-6 on this process. When rCLIP2 was pre-incubated with serpin-1a or serpin-6, the cleavage of proSpz1 was reduced, resulting in more proSpz1 remaining in the culture than in the control group (Fig 4F). Subsequently, protein samples from each group were added to the BmN cell culture medium; the cultured BmN cells were collected 24 h later for RNA extraction. RT-qPCR results showed that the expression of *gloverin2* was significantly increased in BmN cells when an incubation mixture of rCLIP2 and proSpz1 was added. In contrast, the expression of *gloverin2* significantly decreased when serpin-1a or serpin-6 inhibited the cleavage of proSpz1 by CLIP2 (Fig 4G). Hence, serpin-1a and serpin-6 might jointly regulate the activity of CLIP2 to modulate AMP production in silkworms under different physiological conditions.

### Analysis of the synergistic pattern employed by serpin-1a and serpin-6 to regulate CLIP2 activity

**Fig 5:**
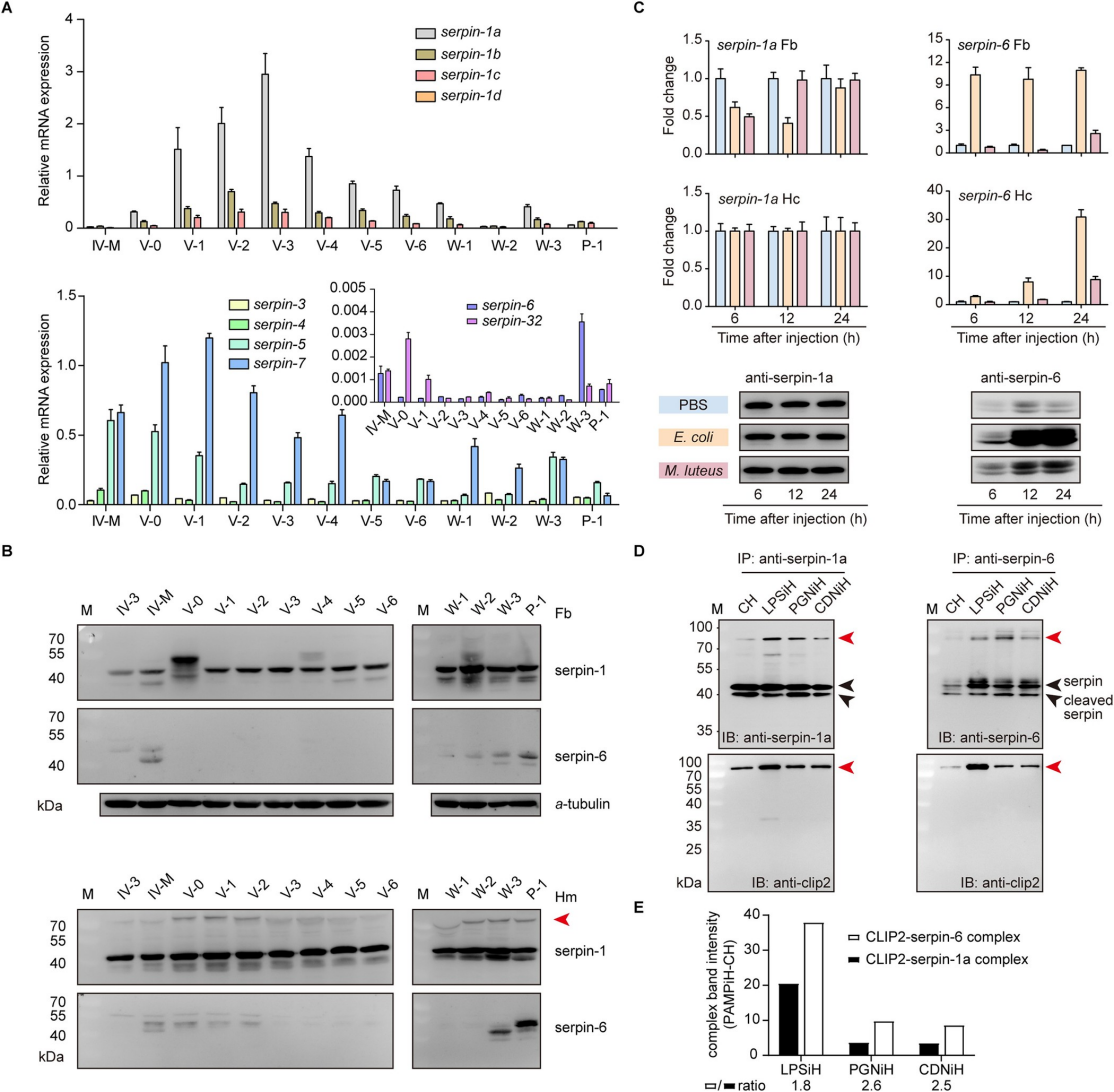
Expression patterns and synergistic regulation analysis of serpin-1a and serpin-6. *B*. *mori* serpin-1 and -6 mRNA and protein levels in in fat body (A) and hemolymph (B) from different developmental stages. The fat body and hemolymph from day 3 of the fourth-instar larvae to day 1 of the pupal stage were collected for RNA isolation and immunoblotting. The transcript levels of serpin-1, -3 to -7, and -32 in the fat body were measured by RT-qPCR (A). Error bars represent mean ± SD (n = 3). The protein abundances of serpin-1 and -6 were detected in the fat body and hemolymph by immunoblotting using antibodies against serpin-1a and -6 (B). Since serpin-1a antibodies can recognize all variants of serpin-1, the detection of serpin-1a protein expression in silkworm fat body and hemolymph is labeled as serpin-1. *a*-tubulin was used as the reference protein. IV-3: day 3 of fourth-instar larvae; IV-M: molting of fourth-instar larvae; V-0~V-6: days 0–6 of fifth-instar larvae; W-1–W-3: days 1–3 after wandering; P-1: day 1 after pupation. The red arrow indicates the potential serpin-protease complex. (C) Effects of *Escherichia coli* and *Micrococcus luteus* on the expression of serpin-1 and -6 in silkworm immune-related tissues. The transcript levels of serpin-1a and -6 in the fat body and hemocyte were measured by RT-qPCR (*upper* and *middle*). The protein abundances of serpin-1 and -6 in hemolymph detected by immunoblotting using antibodies against serpin-1a and -6 (*lower*). (D) Immunoprecipitated purified serpin and associated proteins were subjected to SDS-PAGE and detected by immunoblotting (IB) using antibodies against serpin-1a (*upper left*), serpin-6 (*upper right*), and CLIP2 (*lower*). CH: control hemolymph; LPSiH: the hemolymph induced by lipopolysaccharide (LPS); PGNiH: the hemolymph induced by peptidoglycan (PGN); CDNiH: the hemolymph induced by curdlan (CDN). M: protein molecular weight marker. Red arrows indicate the CLIP2-serpin complex. (E) Intensity of the CLIP2-serpin complex detected by antibodies against CLIP2 (D *lower*) was analyzed using ImageJ. The remaining value after subtracting the gray value of the CH group from the gray value of the PAMPiH (PAMP induced hemolymph) group is used for histogram plotting to compare the intensity of the complex formed between CLIP2 and serpin-1a or -6 under pathological conditions.

In the LC-MS/MS results of the immunoprecipitation complex, serpin-1 was detected in the complex of uninduced and induced hemolymph, whereas serpin-6 was only detected in the complex of induced hemolymph (Fig 4B), suggesting that it may be involved in the regulation of CLIP2 activity under different physiological conditions. To test this hypothesis, we investigated the expression patterns of serpin-1, serpin-3 to serpin-7, and serpin-32 in silkworm immune tissues. Developmental expression profiles in the fat body from the fourth-instar molting stage to day 1 of the pupal stage showed that the expression levels of *serpin-1a* and *serpin-7* were the highest, initially increasing and then decreasing, whereas the expression levels of *serpin-1b*, *serpin-1c* and *serpin-5* were slightly lower, with trends in expression levels similar to those of *serpin-1a*. Meanwhile, the expression levels of *serpin-3* and *serpin-4* were much lower, and those of *serpin-1d*, *serpin-6* and *serpin-32* were the lowest, only expressed in the molting, newly molted, and wandering stages (Fig 5A). Next, the protein levels of serpin-1 and serpin-6 in the fat body and hemolymph were detected by immunoblot analysis. The results showed that serpin-1 protein continued to be present in high abundance in the fat body and hemolymph from day 3 of the fourth-instar larvae to day 1 of the pupal stage, whereas serpin-6 protein only existed in low abundance in the fat body or hemolymph of the fourth-instar molting stage, the early fifth-instar stage, and before and after pupation (Fig 5B). In addition, the serpin-1a antibody recognized a lagging band at a position > 70 kDa in the hemolymph (Fig 5B), which is consistent with the detection results of the CLIP2 antibody (Fig 1B), suggesting that this band is a covalent complex formed by serpin-1a and CLIP2. These results indicate that serpin-1a, with high abundance in the hemolymph, may function as the primary regulator of CLIP2 activity under normal developmental conditions, whereas serpin-6 likely has a secondary role, given its extremely low abundance.

The induction patterns of serpins in silkworm immune tissues were further examined following PAMP and bacterial treatment. RT-qPCR results showed that the expression of the Toll pathway extracellular ligand *Spz1* and AMP genes *gloverin1*, *gloverin2*, *cecropinB*, and *moricin2* were significantly upregulated in the fat body after PAMP stimulation (S4A Fig), indicating that these PAMPs activated the immune pathway in the silkworm. Further detection of *serpin* expression levels in the fat body after PAMPs stimulation revealed that the induced expression levels of *serpin-5* and *serpin-6* were the most significant, followed by *serpin-3* and *serpin-4*, whereas the expression levels of *serpin-1a*, *-1b*, *-1c*, and *serpin-7* remained relatively constant (S4B Fig).

We then detected the expression changes of serpin-1a and serpin-6 in the fat body, hemocytes, and hemolymph after treatment with *E*. *coli* and *M*. *luteus* for 6, 12, and 24 h. Compared to the control, the expression of *serpin-1a* first decreased and then recovered to the same level as the control in the fat body while remaining unchanged in hemocytes. Meanwhile, the expression of *serpin-6* was strongly induced in the fat body and hemocytes, particularly after *E*. *coli* injection (Fig 5C). Immunoblot analysis showed that serpin-1 levels were not significantly impacted in the hemolymph 6–24 h after the immune challenge, whereas serpin-6 levels increased significantly, which was consistent with the mRNA level change (Fig 5C). These results indicate that the expression levels of serpin-1a, -1b, -1c, and -7 were not induced by immune challenge, whereas those of serpin-3 to -6 were significantly induced upon immune challenge. Expression analysis showed that the expression levels of serpins in silkworm immune tissues vary greatly under different physiological conditions. The synergistic expression of constitutive serpin (serpin-1a and serpin-7) and inducible serpin (serpin-3 to -6) is conducive to the precise regulation of silkworm immune level under physiological and pathological conditions.

These results led us to speculate that the activity of CLIP2 is primarily regulated by serpin-1a, which is highly abundant in the hemolymph during normal development. However, after encountering an immune challenge, the induced serpin-6 assists serpin-1a in regulating CLIP2 activity. To test this hypothesis, we analyzed the interaction patterns of serpin-1a and serpin-6 with CLIP2 *in vivo* using immunoprecipitation. LPS, PGN, and CDN were injected into the larval hemocoel on day 2 of the fifth-instar; the hemolymph of each treatment group was collected 24 h later for immunoprecipitation. Hemolymph collected from naïve *B*. *mori* larvae was used as a control. Co-immunoprecipitation with serpin-1 or -6 antibodies allowed us to isolate serpin and inhibitor-enzyme complexes from the hemolymph sample. Immunoblot results showed that serpin-1a forms a complex with CLIP2 in the hemolymph during normal development and immune responses, whereas serpin-6 only forms an obvious complex with CLIP2 during immune responses (Fig 5D). In addition, serpin-CLIP2 complexes were markedly increased in the induced hemolymph after PAMPs stimulation. The remaining value after subtracting the gray value of the CH (control hemolymph) group from the gray value of the PAMPiH (PAMP induced hemolymph) group is used to compare the intensity of the complex formed between CLIP2 and serpin-1a or -6 under pathological conditions. The results showed that the intensity of the CLIP2-serpin-6 complex was about twice that of the CLIP2-serpin-1a complex after PAMPs stimulation (Fig 5E). This suggests that serpin-6 has higher affinity to CLIP2 than serpin-1a in the induced hemolymph. These results confirmed our hypothesis that serpin-1a and serpin-6 synergistically regulate the activity of clip2 in the silkworm hemolymph under different physiological conditions.

### Effect of knocking out of *serpin-1* on silkworm immune response

To further verify the regulatory mechanism of CLIP2 activity in silkworms, we performed CRISPR/Cas9-mediated genome editing to knock out *serpin-1*. To obtain homozygous mutants with *serpin-1* loss of function, the chimeric mutants of *serpin-1* were first crossed with wild-type individuals; subsequently, the screened heterozygous mutants with the 4 bp deletion were self-crossed, and the homozygous mutants were screened from the offspring (S5 Fig).

**Fig 6:**
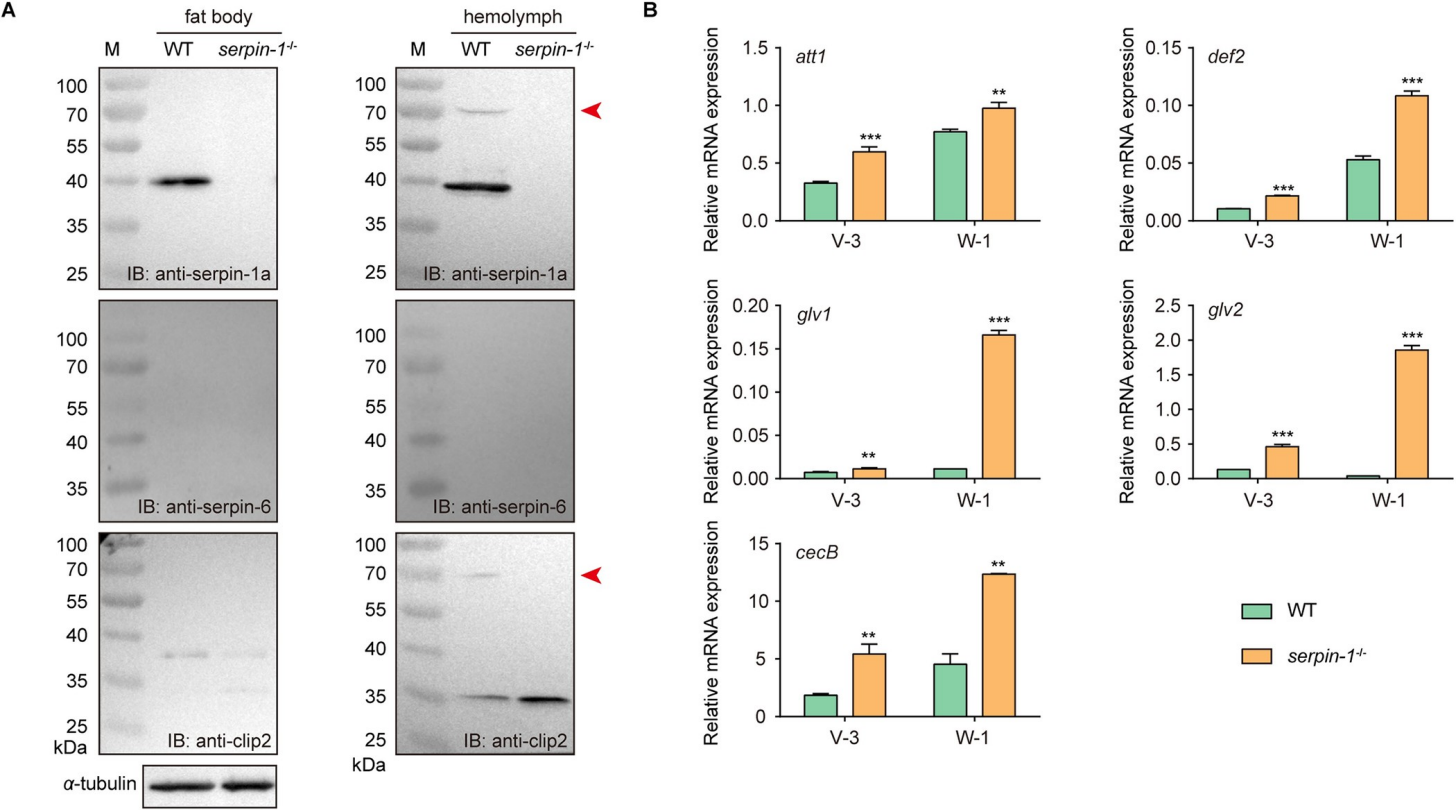
Effect of knockout *serpin-1* on the background expression of antimicrobial peptide genes. (A) Serpin-1, -6, and CLIP2 protein levels in the fat body and hemolymph of wild-type (WT) and *serpin-1* knockout (*serpin-1*^*-/-*^) individuals on day 3 of the fifth-instar. The protein levels of serpin-1, -6 and CLIP2 were detected by immunoblotting (IB) using antibodies against serpin-1a (*upper*), -6 (*middle*) and CLIP2 (*lower*). *a*-tubulin was used as the reference protein. M: protein molecular weight marker. Red arrows indicate the CLIP2-serpin-1a complex. (B) The background expression levels of antibacterial peptide genes in the fat body of WT and *serpin-1*^*-/-*^ individuals on day 3 of the fifth-instar and day 1 of the wandering stage detected using RT-qPCR. Error bars represent mean ± SD (*n* = 3). **P < 0.01; ***P < 0.001.

Immunoblotting results showed that within the fat body and hemolymph of wild-type (WT) and *serpin-1* KO individuals on day 3 of the fifth-instar, serpin-1 was only detected in the fat body and hemolymph of WT individuals and formed a complex with CLIP2 in the hemolymph. Meanwhile, the CLIP2 content increased in the hemolymph of *serpin-1* KO individuals, whereas serpin-6 was not detected in the fat body and hemolymph of WT or *serpin-1* KO individuals due to its low content (Fig 6A). RT-qPCR further revealed that the expression of *attacin1*, *cecropinB*, *defensin2*, *gloverin1*, and *gloverin2* was significantly upregulated in the fat bodies of *serpin-1* KO individuals compared with WT individuals, and the changes were more significant during the wandering stage (Fig 6B). Hence, the abundance of CLIP2 increased and the background expression level of AMPs improved following *serpin-1* knockout.

**Fig 7:**
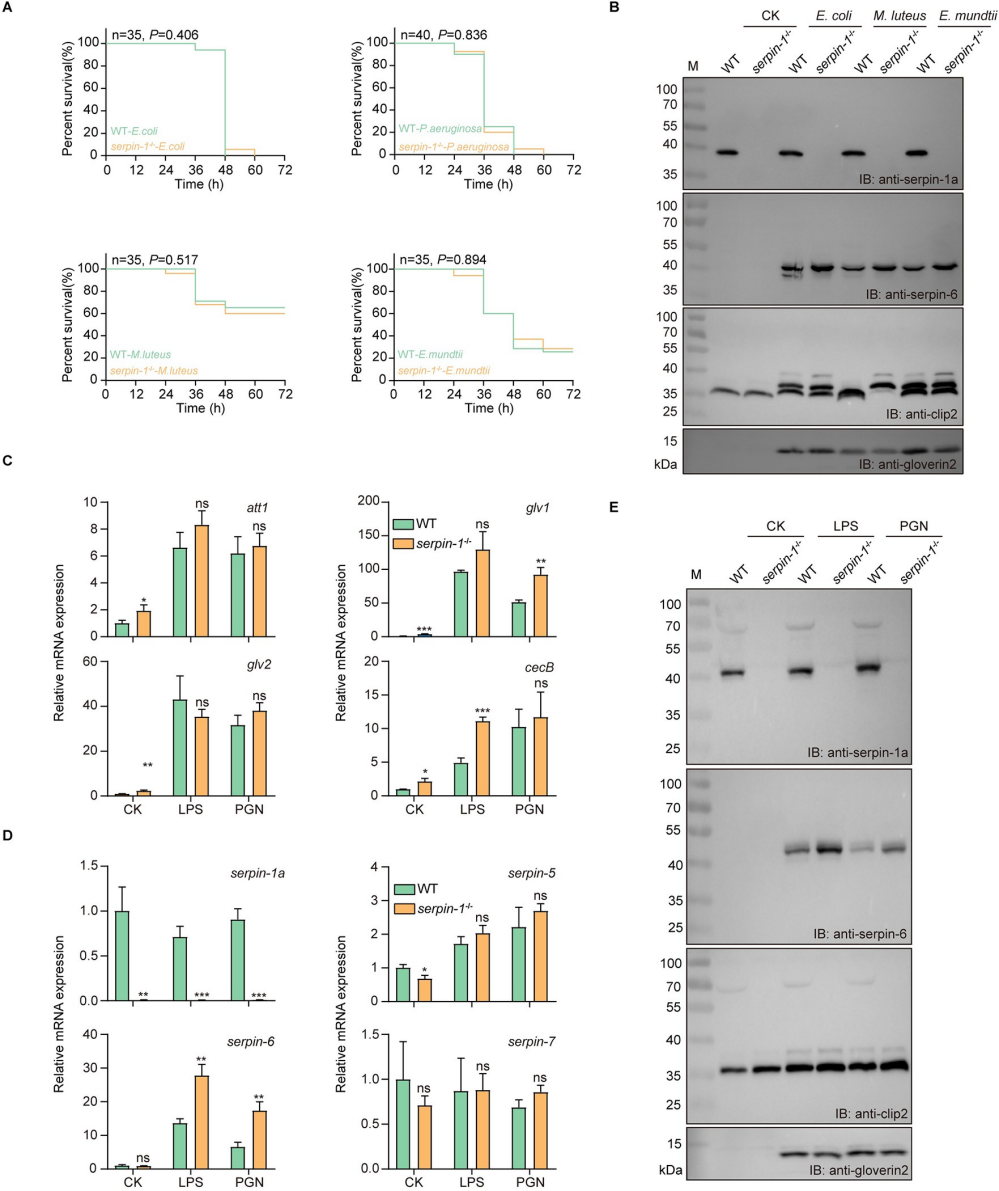
Effects of *serpin-1* knockout on serpin-6 expression and immune homeostasis in silkworm under immune challenge. (A) Wild-type (WT) and *serpin-1* knockout (*serpin-1*^*-/-*^) individuals were respectively injected with *Escherichia coli*, *Pseudomonas aeruginosa*, *Micrococcus luteus*, or *Enterococcus mundtii*; survival curves were plotted until 72 h post-infection. Statistical analysis between WT and *serpin-1*^*-/-*^ groups were calculated using the log-rank test. (B) The hemolymph of each group was collected at 24 h post-injection to detect the levels of serpin-1, serpin-6, CLIP2 and gloverin2 proteins by immunoblotting (IB). WT and *serpin-1*^*-/-*^ individuals were injected with 5 μL PBS (CK), 2.5 μg lipopolysaccharide (LPS) or peptidoglycan (PGN). At 18 h post-injection, the fat body of each group was collected to detect the transcript levels of *serpins* (C) and antibacterial peptide genes (D) by RT-qPCR; the hemolymph of each group was collected to detect the protein levels of serpins, CLIP2 and gloverin2 by immunoblotting (E). M: protein molecular weight marker. Error bars represent mean ± SD (*n* = 3). *P < 0.05, **P < 0.01, and ***P < 0.001.

To determine if the increased AMP levels observed in *serpin-1* KO individuals correlate with increased resistance to pathogens, we examined whether *serpin-1* KO individuals were resistant to gram-negative bacteria (*E*. *coli* and *P*. *aeruginosa*) or gram-positive bacteria (*M*. *luteus* and *E*. *mundtii*) by assessing their survival within 72 h of bacterial infection. The viability of *serpin-1* KO individuals did not differ significantly from WT individuals infected with under the infection of *E*. *coli* (*P* = 0.406), *P*. *aeruginosa* (*P* = 0.836), *M*. *luteus* (*P* = 0.517), or *E*. *mundtii* (*P* = 0.894) (Fig 7A). Further immunoblotting revealed that the expression of serpin-6, CLIP2, and gloverin2 in the hemolymph of the pathogen-infected group was upregulated compared with the control group, and the upregulated level of serpin-6 in *serpin-1* KO individuals was significantly higher than that in WT individuals (Fig 7B). These results indicate that knocking out *serpin-1* did not affect the background expression level of serpin-6 under normal conditions, however, promoted induced expression of serpin-6 under pathogen infection. Hence, serpin-1 and serpin-6 may synergistically regulate the immune homeostasis of the Toll pathway under normal and pathogenic conditions.

To further confirm the effects of *serpin-1* KO on serpin-6 expression and immune homeostasis, we examined the expression levels of serpins, CLIP2, and AMPs in WT and *serpin-1* KO individuals after LPS or PGN stimulation. RT-qPCR results showed that the expression changes of AMP genes in the fat bodies of WT and *serpin-1* KO were not obvious after LPS or PGN stimulation; only *gloverin1* after PGN stimulation and *cecropinB* after LPS stimulation were upregulated in *serpin-1* KO individuals compared to WT individuals (Fig 7C). Further detection of *serpins* expression after LPS and PGN stimulation showed that the expression of *serpin-6* was significantly upregulated (> 2-fold) in the fat body of *serpin-1* KO individuals compared to WT individuals after LPS or PGN stimulation, whereas no difference was observed in *serpin-5* or *serpin-7* expression (Fig 7D). Immunoblotting showed that the expression of serpin-6, CLIP2, and gloverin2 was upregulated in the hemolymph of the LPS- or PGN-infected group compared with the control group, and the upregulated level of serpin-6 in *serpin-1* KO individuals was significantly higher than that in WT individuals, consistent with the transcription levels results (Fig 7E). Hence, immune stimulation following *serpin-1* KO caused compensatory expression of serpin-6, which in turn regulated CLIP2 activity and AMP expression.

## Discussion

Here, we characterize the immune function of the terminal protease CLIP2 of the Toll extracellular signaling cascade in the silkworm and systematically analyze the physiological mechanism by serpin-1a and serpin-6 in regulating the immune homeostasis of the Toll pathway through synergistically inhibiting CLIP2.

Invertebrate Tolls and vertebrate Toll-like receptors (TLRs), have a crucial role in the detection of microbial infection in insects and mammals, respectively [47,48]. In mammals, the activation of the Toll pathway is initiated by TLRs binding to various PAMPs, whereas in insects, its activation requires the binding of the cytokine Spz to the Toll receptor [49–51]. Insect proSpz is secreted as an inactive precursor and requires proteolytic processing at a specific site to produce an active ligand [16,44,52,53]. Our previous studies showed that silkworm CLIP2 is evolutionarily clustered in the same branch as SPE and HP8, which function as proSpz-activating proteases in *D*. *melanogaster* and *M*. *sexta* [31]. In the present study, our data indicated that CLIP2 directly cleaves the proSpz1 protein to generate an active ligand that activates the expression of AMPs in silkworms.

The fat body, hemocytes, and circulating hemolymph are important immune tissues for insects [1,54]. *Drosophila* SPE and *Manduca* HP8 are expressed constitutively in the fat body and hemocytes during the larval stages; the former is rapidly upregulated in response to immune challenge, while the latter is not upregulated following bacterial or β-1,3-glucan curdlan stimulation [15,55]. We found that silkworm CLIP2 is constitutively present in larval hemolymph, and its expression is significantly upregulated after bacterial or PAMP stimulation, similar to the expression pattern of *Drosophila* SPE. However, *Drosophila* and *Manduca Spz1* mRNA primarily expressed in hemocytes and increased after immune challenge, whereas *Bombyx Spz1* mRNA was most abundant in the midgut and fat body, and was also upregulated after microbial stimulation [16,56–58]. Our results show that the transcriptional expression of *CLIP2* and *Spz1* in the fat body exhibit a staggered high expression pattern, which may be related to background activation of the silkworm Toll pathway.

Moreover, detection of the effector molecule gloverin2 showed that its background expression level significantly correlated with CLIP2 and Spz1 expression in silkworm fat bodies and hemolymph. This implies that as the final step in the extracellular protease cascade of the Toll pathway, the cleavage of proSpz by the terminal protease may be tightly regulated to ensure correct transmission and timely termination of extracellular signals.

Indeed, our *in vitro* cleavage assay demonstrated that CLIP2 can process proSpz1 to release an Spz1-C fragment. Previous studies have cloned and expressed the truncated form of *B*. *mori* Spz1, to mimic the active ligand [57]. The molecular weight of Spz1-C matched the size of the truncated form of *B*. *mori* Spz1 and was similar to that of the proSpz-1A product cleaved by *M*. *sexta* HP8 [16], indicating that the Spz1-C fragment produced by CLIP2 may be an active ligand in the silkworm Toll pathway. *In vivo* injection analyses further indicated that the Spz1-C fragment strongly stimulated AMP expression in the silkworm fat body, which is consistent with the results obtained after injection of the active Spz of *M*. *sexta* and *B*. *mori* [16,57], indicating that the Spz1-C fragment is biologically active *in vivo*. The injected proSpz1 also induced small increases in AMP expression in the silkworm fat body, possibly due to its cleavage by endogenous CLIP2 or other proteases. In *M*. *sexta*, injection of activated HP8 results in increased expression levels of AMPs in the fat body and antimicrobial activity in the hemolymph [15]. During the wandering stage, with high expression levels of endogenous *Spz1*, injection of CLIP2 also increased the expression of AMPs and induced strong antibacterial activity in the hemolymph. These results indicate that CLIP2 is responsible for processing proSpz to activate the Toll pathway in silkworms.

**Fig 8:**
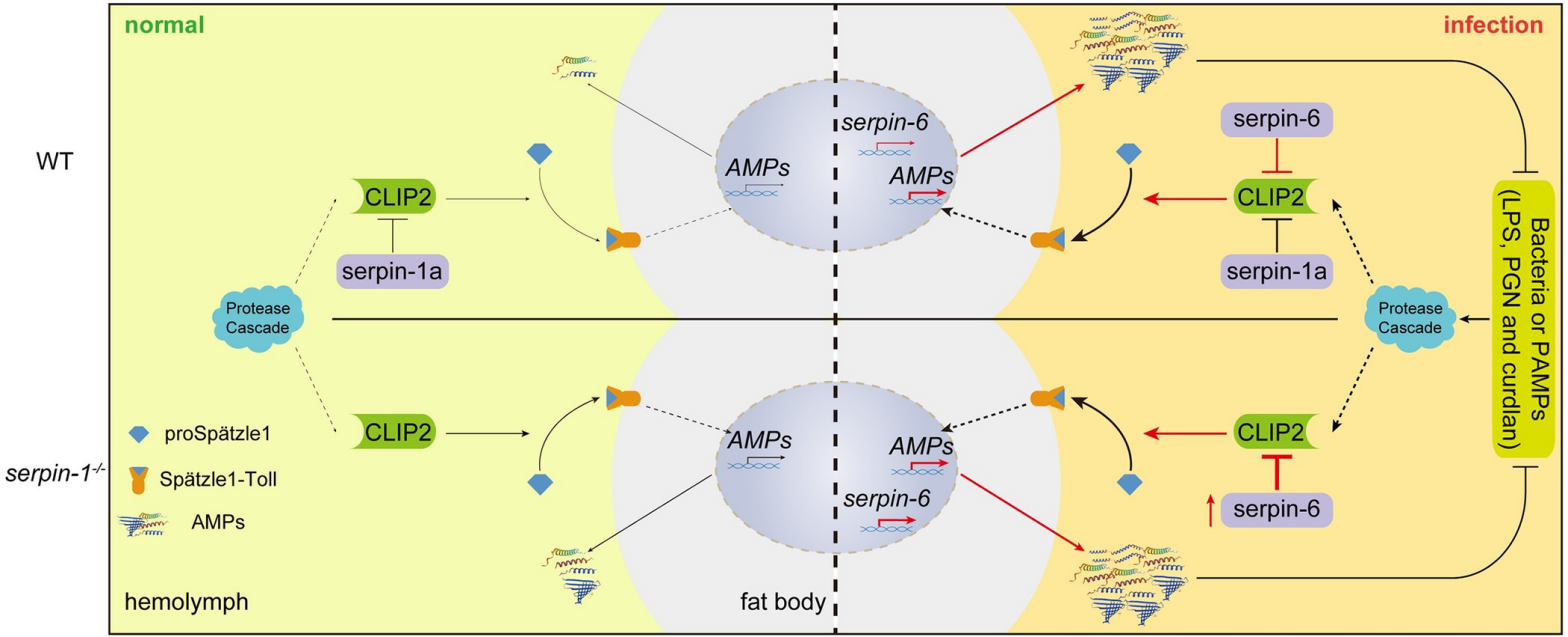
Schematic diagram representing the synergistic mechanism of serpin-1a and serpin-6 in the regulation of silkworm Toll pathway homeostasis under normal and infection conditions. Activation of the silkworm immune cascade CLIP2-Spz1 is co-regulated by serpin-1a and serpin-6: (1) under normal conditions, the activity of CLIP2 is regulated primarily by the constitutively expressed serpin-1a in hemolymph; Toll pathway activation is maintained at background levels in the fat body. (2) under pathogen infection conditions, the expression and activation of CLIP2 in the hemolymph increases, and serpin-6 expression is induced to assist serpin-1a in regulating CLIP2 activity, effectively avoiding excessive Toll pathway activation, and maintaining immune homeostasis. PAMPs, pathogen-associated molecular patterns; LPS, lipopolysaccharide; PGN, peptidoglycan; AMPs, antimicrobial peptides. The thickness of the lines indicates the intensity of signal transmission or the level of gene expression.

The balance between activation and inhibition of the extracellular protease cascade must be strictly regulated to maintain immune homeostasis and avoid damage to the host [20,59]. Previous studies have shown that serpins regulate insect innate immunity by forming covalent complexes with target proteases to inhibit extracellular protease cascade signaling [18,19,23–25,33,60]. Three *T*. *molitor* serpins have been characterized, each target a specific member of the Toll cascade-activating SPs and block the activation of proSpz in a reconstituted pathway *in vitro* [19]. Unlike specific SP-serpin pairs in *T*. *molitor*, members of *M*. *sexta* Toll cascade-activating SPs typically form covalent complexes with multiple serpins. For example, HP5 forms 75 kDa complexes with serpin-1A, serpin-1J and serpin-4 [11]; proHP1 in an active conformation forms 90 kDa covalent complexes with serpin-1, serpin-4, serpin-9, and serpin-13 [61]; meanwhile, HP8 forms an SDS-stable complex with serpin-1J, serpin-3, and serpin-6 [24,25,62]. Previous biochemical studies have indicated that each protease in the extracellular SP cascade of the insect Toll pathway is regulated by one or more specific serpins, demonstrating an elaborate regulatory mechanism of insect immune defense reactions. In the present study, we found that serpin-1a and serpin-6, targeted the Toll cascade-activating terminal protease CLIP2, functioning negative regulators to synergistically maintain immune homeostasis of the silkworm Toll pathway under physiological and pathological conditions. Both serpin-1a and serpin-6 formed SDS-stable complexes with active CLIP2. However, the reaction with serpin-6 was more rapid and indicates that serpin-6 is a more efficient inhibitor of CLIP2. Moreover, our data demonstrated that the activation of the silkworm immune cascade CLIP2-Spz1 was co-regulated by serpin-1a and serpin-6. That is, during the normal development of silkworms, the activity of CLIP2 is primarily regulated by serpin-1a with in the hemolymph, while activation of the Toll pathway is maintained at the background level (Fig 8). However, when pathogens invade silkworms, an increase in CLIP2 expression and activation is induced with in the hemolymph. The induced serpin-6 cooperatively assists serpin-1a in the regulation of CLIP2 activity, avoiding excessive activation of the Toll pathway, and maintaining immune homeostasis (Fig 8). Collectively, these findings highlight the importance of the precise regulation of Toll cascade-activating terminal proteases in maintaining insect immune homeostasis.

In *T*. *molitor*, the terminal protease SPE is regulated by serpin-48 and the N-terminal serpin domain of the twin-domain serpin serpin-93 [19,23]. Similar to silkworm serpin-1a, *T*. *molitor* serpin-48 is constitutively expressed in larval hemolymph, and is not affected by β-1,3-glucan stimulation. In *M*. *sexta*, the terminal protease HP8 is regulated by serpin-1J and serpin-6 [24,25]; serpin-1 encodes 12 variants with differences in the RCL sequence, in which serpin-1J regulates proPO system and Toll pathway activation by inhibiting proPO-activating protease-3 and HP8, respectively [24,63,64]. The expression level of *serpin-1J* in the larval fat body is lower than *serpin-1A*, *1B*, *1E*, and *1H* and immune challenge does not increase the transcript levels of any variants [65,66]. As the orthologous gene of *M*. *sexta* serpin-1, *B*. *mori* serpin-1 encodes four variants to produce three serpins with difference in the RCL sequence (serpin-1a–d or sw-AT-1–4) [31,67]. Our data showed that *serpin-1a* was significantly more highly expressed compared with the other three variants, and its expression was not stimulated by immune challenge in the fat body. Although both *M*. *sexta* serpin-1J and *B*. *mori* serpin-1a can regulate the expression of AMPs, serpin-1a does not inhibit proPO activity or melanization of silkworm hemolymph (S6A and S6B Fig). Meanwhile, in the current study, knocking out *serpin-1* resulted in an increase in CLIP2 abundance and background AMP expression in the absence of immune challenge. *In vivo* experiments further showed that injection of recombinant serpin-1a into *B*. *mori* larvae led to significantly diminished expression of AMPs in the fat body (S6C Fig). These results indicate that serpin-1a is a physiologically important regulator of CLIP2 and participates in the regulation of silkworm immune homeostasis. Similarly, in *D*. *melanogaster*, lack of serpin-43Ac or serpin-1 leads to constitutive expression of the Toll-mediated antifungal peptide drosomycin in the absence of infection [21,22]. However, unlike serpin-1a, serpin-43Ac and serpin-1 act upstream of the Toll cascade-activating terminal protease SPE. In addition, *serpin-43Ac* and *serpin-1* are immune-responsive genes, and their expression is positively controlled by the Toll pathway [21,22], whereas *serpin-1a* is an immune unresponsive gene whose expression is significantly downregulated after starvation (S6D Fig). Compared to serpin-1J, serpin-43Ac, and serpin-1, our data highlight the unique expression patterns and physiological functions of serpin-1a in insect immune responses. Another orthologous pair, *M*. *sexta* and *B*. *mori* serpin-6, exhibit similar immunoregulatory functions, inhibiting the activation of the proPO system and AMP expression [25,34]. In both species, the expression of serpin-6 exhibited low background levels in the larval fat body, which increased significantly following immune challenge. Hence, our data indicate that serpin-6, an acute-phase protein, synergizes with serpin-1a to regulate the expression of AMPs and maintain immune homeostasis under pathogen challenge. The synergistic pattern of serpin-1a and serpin-6 in regulating the immune homeostasis of the Toll pathway may also be an important way for insects to maintain the balance between development and immunity. These studies demonstrate that the control of Toll cascade-activating signaling is far more complex and precisely regulated than previously thought.

The viability of *serpin-1* KO individuals after immune challenge with bacteria did not differ from that of WT individuals, which contradicted the expectation that AMP expression will become increased in the absence of immune challenge. Further analysis revealed that immune stimulation after knocking out *serpin-1* induced compensatory expression of serpin-6, resulting in increased CLIP2 and AMP expression in *serpin-1* KO individuals, consistent with those in WT individuals. These results not only explain the survival rate of *serpin-1* KO individuals under pathogen infection but also further demonstrate the specific synergistic relationship between serpin-1a and serpin-6 in silkworm immune responses. However, the mechanism by which serpin-6 expression is induced by immune stimulation remains unclear.

Immune-responsive serpin expression is typically upregulated by immune challenge, indicating an inducible negative feedback inhibition mechanism. In *D*. *melanogaster*, *T*. *molitor*, and *Aedes aegypti*, the expression of immune-responsive serpin-43Ac, serpin-1, serpin-2, serpin-40, and serpin-55 is under the positive control of the Toll pathway [19,21,22,68]. Although it has been shown that, in *Anopheles gambiae*, expression of the acute-phase protein serpin-6 is regulated by the LPS-induced TNFα transcription factor (LITAF), it is currently unclear how *LITAF* expression is regulated [69]. Our data showed that knocking out *serpin-1* promoted the expression of AMPs in the absence of immune stimulation, without impacting *serpin-6* expression. This suggests that the expression of *serpin-6* may not be regulated by negative feedback from the Toll pathway, but rather by other pathways or factors, such as the LITAF-like transcription factor. In addition, organisms have developed various genetic buffering systems to maintain normal growth and development despite genetic or environmental perturbations, including functional redundancy, rewiring of genetic networks and genetic compensation responses [70–72]. We speculate that the expression of *serpin-6* in *serpin-1* KO individuals was significantly higher than that in WT individuals under immune stimulation, which may represent a passive functional compensation mechanism to maintain immune homeostasis. Therefore, in-depth analysis of the induced expression of serpin-6 and its functional compensation mechanism for the serpin-1 mutation will help to better understand the role of serpin-1a and serpin-6 in Toll pathway immune homeostasis. Future research should certainly further investigate the molecular mechanism of serpin-6 passive compensation for serpin-1a.

Collectively, our results revealed the role of the synergistic regulation of serpin-1a and serpin-6 in the maintenance of silkworm immune homeostasis. Our biochemical and genetic studies demonstrated that serpin-1a and serpin-6 regulate Toll pathway immune homeostasis by synergistically inhibiting CLIP2 under physiological and pathogenic conditions. Hence, this study provides novel insights into the precise regulation of Toll cascade-activating signaling in the innate immune response of silkworm and highlight the importance and complexity of regulating insect immune homeostasis.

## Materials and methods

### Materials and sample collection

The *B*. *mori* strains Dazao and N4 were reared on fresh mulberry leaves under a 12 h light/12 h dark photoperiod at 25°C and 75 ± 5% relative humidity. *Escherichia coli*, *Pseudomonas aeruginosa*, *Micrococcus luteus*, *Enterococcus mundtii* and *Pichia pastoris* strain GS115 were maintained at the Biological Science Research Center at the Southwest University of China. PAMPs (Sigma-Aldrich, St. Louis, MO, USA), including *E*. *coli* LPS, *M*. *luteus* PGN, and *Alcaligenes faecalis* CDN, were diluted in PBS to a final concentration of 0.5 μg/μL. The polypeptide substrate N-acetyl-Ile-Glu-Ala-Arg-*p*-nitroanilide acetate (IEAR-pNA) was synthesized by GenScript. Hemolymph samples used for immunoblotting, co-immunoprecipitation and antibacterial activity assays were centrifuged at 4°C, 5,000 ×g for 15 min to remove the hemocytes.

To detect gene expression patterns, the fat body and hemolymph from day 3 of the fourth-instar larvae to day 1 of the pupal stage were collected for RNA isolation and immunoblotting. On day 2 of the fifth-instar, larvae were fasted without food for 12 h to ensure hunger, and then chilled on ice for 10 min. For microorganism induction, each group was injected with 5 μL of PBS (control group), a gram-positive bacterium (dried *M*. *luteus*, 0.5 μg/μL), and a gram-negative bacterium (killed *E*. *coli*, 2 × 10^6^ cells/μL), respectively. At 6, 12, and 24 h post-injection, the fat body, hemocytes, and hemolymph of the silkworm were collected for RNA isolation or immunoblotting. For PAMP induction, each group was injected with 5 μL of PBS (control group), LPS, PGN, or CDN. Twelve hours post-injection, the fat bodies of the silkworms were collected for RNA isolation.

### Recombinant protein purification and activity analysis

To achieve recombinant protein expression of proCLIP2, the open reading frame of proCLIP2 without the signal peptide was subcloned into the pPICZαA vector with *Mfe*I and *Not*I. At the predicted activation site of proCLIP2 a mutation was introduced by changing residues 85–88 from NNDR to IEGR via site-directed mutagenesis. The IEGR is a cleavage site for factor Xa protease. This construct was designated proCLIP2_Xa_. The recombinant plasmid pPICZαA-proCLIP2_Xa_ was linearized and electroporated into the competent yeast GS115 cells. Highly productive clones were selected based on bleomycin resistance (1000 μg/mL) and PCR detection. The selected high-productivity clones were cultured in BMGY at 28°C until the optical density at 600 nm (OD_600_) reached 5.0. The BMGY medium was removed after centrifugation, and the cells were resuspended in BMMY medium. After the OD_600_ reached 1.0, 1% (v/v) methanol was added every 24 h to the BMMY medium, and the culture medium supernatant was collected by centrifugation after induction for 72 h for recombinant protein purification. Recombinant proCLIP2_Xa_ was purified using a nickel-nitrilotriacetic acid column (Ni-NTA; GE Healthcare) according to the manufacturer’s instructions.

To generate proSpz1, serpin-1a, and serpin-6 recombinant proteins, the open reading frames of these genes without the signal peptide were subcloned into the pET28a vector. To confirm the P1 site against trypsin of serpin-1a RCL, we constructed and expressed the E329A, R340A and S341A mutants based on previous studies [31,73]. Among them, E329 has been shown to play an important role in the proper folding of serpin-1a RCL [73]. The recombinant plasmids were transformed into *E*. *coli BL21* (DE3) cells. Recombinant protein expression was induced with 0.1 mM isopropyl-β-D-thiogalactoside at 16°C for 20 h or 37°C for 4 h. *E*. *coli* cells were harvested by centrifugation (6,000 *×*g, 20 min) and lysed by sonication in a binding buffer (20 mM Tris, 200 mM NaCl, pH 8.0). After centrifugation (12,000 *×*g, 4°C, 20 min), the recombinant proteins in the supernatant or precipitate were purified using a Ni-NTA column. The purified recombinant proSpz1 inclusion body protein was renatured by dialysis against renaturation buffer (8 M urea, 20 mM Tris-HCl, 100 mM NaCl, 2 mM reduced glutathione, 0.2 mM oxidized glutathione) with decreasing concentrations of urea (6, 4, and 2 M). Each dialysis step was performed at 4°C for 12 h and the final buffer comprised 20 mM PBS (pH 7.4). The refolded supernatant was filtered through a 0.22-μm microporous membrane and subjected to 12% SDS-PAGE. The primers used for vector construction are listed in S2 Table.

The proCLIP2_Xa_ activity assay was performed using IEAR-pNA as a substrate. proCLIP2_Xa_ (0.75 μg) was pre-incubated with factor Xa (1 μg) for 20 min at room temperature. Subsequently, factor Xa (1 μg), proCLIP2_Xa_ (0.75 μg), and pre-incubation solution of both were mixed with 150 μL of 50 μM IEARpNA substrate in 96-well microtiter plates (three replicates each); 20 mM PBS (pH 7.4) was added to a final reaction volume of 200 μL. After incubation at room temperature for 1 h in the dark, the change in absorbance at 405 nm was measured using a multifunctional microplate reader (Promega, Madison, WI, USA), and the relative activity of each group was calculated.

The inhibitory activities of recombinant serpin-1a and recombinant serpin-6 were determined as previously described, using FITC-casein as the target protease substrate [37]. Recombinant serpin protein (10 μg) was incubated with different commercial proteases (1 μg) in 100 μL of assay buffer (100 mM Tris-HCl, pH 7.5) for 30 min at room temperature in 96-well microtiter plates. FITC-casein (10 ng, Thermo Fisher Scientific, USA) in 100 μL assay buffer was added, followed by incubation in the dark for 1 h at room temperature. Substrate hydrolysis was monitored using a multifunctional microplate reader at excitation and emission wavelengths of 485 nm and 535 nm, respectively. Protease inhibition by serpin was assessed using the following formula: inhibition (%) = (1 –residual enzyme activity/enzyme activity without inhibitor) × 100%.

### Antibodies

Polypeptide antibodies against CLIP2 protein were obtained by antigen purification after immunizing New Zealand white rabbits with the synthetic peptide C-ENYDPNDKDQQNDIA-NH2. The specificity of CLIP2 antibody was demonstrated by immunoblot analysis of larval cell-free hemolymph and recombinant proCLIP2_Xa_ (S1A Fig). Polyclonal antibodies against serpin-1a or serpin-6 proteins were obtained by antigen purification after immunization of New Zealand white rabbits with recombinant serpin proteins. Polyclonal antibodies against gloverin2 protein were prepared in the same manner that described in our previous study [74].

### Quantitative real time PCR (RT-qPCR)

Total RNA was extracted from different tissues using TRIzol reagent (Invitrogen, USA), and cDNA was synthesized using TransScript One-Step gDNA Removal and cDNA Synthesis SuperMix (TransGen Biotech, China) according to the manufacturer’s protocol. Using the prepared cDNA as a template, the qTOWER^3^ Real-Time PCR Thermal Cycler System (Analytik Jena, Germany) and SYBR Green Kit (TaKaRa Bio, Japan) were used for RT-PCR according to the manufacturer’s instructions. The housekeeping Eukaryotic translation initiation factor 4A (*eIF4A*) gene of *B*. *mori* was used as an internal reference gene. The relative mRNA expression level of target genes was calculated based on the 2^−△△Ct^ method. The primers used for RT-qPCR analysis are listed in S2 Table.

### SDS-PAGE and immunoblot analysis

Silkworm fat body samples were homogenized in lysis buffer (7 M urea, 4% chaps, and 50 mM dithiothreitol). Protein concentrations in tissue samples were determined using the Bradford assay. For SDS-PAGE analysis, recombinant protein or tissue protein samples (0.5–20 μg) were treated with 5×SDS loading buffer for 5 min at 95°C and then separated using 12% (w/v) SDS-PAGE. Proteins were detected by staining with Coomassie brilliant blue (CBB). For immunoblot analysis, the proteins were electrotransferred onto polyvinylidene difluoride membranes. Membranes were blocked overnight at 4°C with 5% (w/v) dried skim milk in Tris-buffered saline containing 0.1% (v/v) Tween 20 (TBST). The membranes were then incubated with a polyclonal primary antibody against the target protein (diluted 1:10,000) and horseradish peroxidase-labeled goat anti-rabbit IgG as the secondary antibody (diluted 1:20,000; Beyotime, China). Signals were detected using the Super-Signal West Femto Maximum Sensitivity Substrate (Thermo Scientific, Waltham, MA, USA). *a*-tubulin (GenBank accession no. AB072304) was used as the reference protein in fat body.

### *In vitro* incubation assay

*In vitro* incubation analysis of recombinant proteins involved in the interactions between CLIP2, Spz1, and serpins was performed as follows.

Analysis of CLIP2 and proSpz1: 0.25 μg of rCLIP2 and 1.0 μg of proSpz1 were incubated for 30 min at room temperature, and then 0.25 of μg rCLIP2, 1.0 μg of proSpz1, and the incubation mixture were subjected to SDS-PAGE; and the molecular weight changes of proSpz1 were detected by CBB staining and immunoblot analysis.

Incubation analysis of CLIP2 and serpins: 0.25 μg of rCLIP2 and corresponding serpin were mixed and incubated at room temperature for 5 min according to the molar mass ratio of 1:5, and then an equal mass of rCLIP2, serpins and the incubation mixture were subjected to SDS-PAGE. Immunoblot analysis was performed using antibodies against CLIP2, serpin-1a, serpin-6, and the His-tag to determine whether rCLIP2 forms a covalent complex with serpins. As an irreversible serine protease inhibitor, phenylmethylsulfonyl fluoride (PMSF), which specifically recognize and sulfonate the active site serine residues that determine the activity of serine proteases, is used to block the binding between rCLIP2 and serpin in partial incubation experiments.

### *In vivo* injection and AMP expression analysis

Based on the expression changes of *CLIP2* and *Spz1* in the silkworm fat body and hemolymph, an *in vivo* injection experiment for recombinant proteins was carried out on day 2 of the fifth-instar (low Spz1 expression level) and day 1 of the wandering stage (high Spz1 expression level).

To analyze the physiological function of CLIP2-proSpz1, day 2 of the fifth-instar larvae were injected with 0.25 μg of rCLIP2, 1 μg of proSpz1, and incubation mixture (0.25 μg rCLIP2 and 1 μg proSpz1) or an equal volume of PBS. Twelve hours after injection, the fat bodies and hemolymph of each group were collected to detect AMP expression levels. Furthermore, larvae at day 1 of the wandering stage were injected with 1 μg of rCLIP2 or 1 μg of BSA. At 12 and 24 h after injection, the fat bodies and hemolymph of each group were collected to detect the expression levels of AMPs and analyze the antibacterial activity of the hemolymph. The antimicrobial activity assays were performed as described by Wang *et al*. [75] and Kausar *et al*. [76]. The primers used for RT-qPCR analysis are listed in S2 Table.

### Co-immunoprecipitation (co-IP) and LC-MS/MS

To identify the regulators of CLIP2, co-immunoprecipitation was performed using polyclonal antibodies against CLIP2. First, 100 μL of Dynabead Protein G (Thermo Fisher, USA) were incubated with 10 μg of polyclonal antibodies against CLIP2 in 200 μL of PBST (20 mM PBS, pH 7.4, 0.02% Tween-20) for 30 min at room temperature. After the bead-Ab complexes were collected by Magnet, they were washed thrice times with 200 μL of conjugation buffer (20 mM Na_3_PO_4_, 150 mM NaCl, pH 8.0), resuspended in 250 μL of conjugation buffer with 5 mM Bis(sulfosuccinimidyl) suberate (BS^3^ crosslinker, Thermo Fisher, USA). After rotating incubation at room temperature for 30min, 12.5 μL of quenching buffer (1 M Tris-HCl, pH 7.5) was added to terminate the crosslinking reaction of the beads and polyclonal antibodies against CLIP2. After the cross-linked bead-Ab complexes were collected, they were washed thrice times with 200 μL of PBST, resuspended in 400 μL of PBS, and stored at 4°C until use.

Hemolymph samples were prepared as previously described [62,77] with slight modifications. Briefly, hemolymph (20 mL) were collected from normal larvae (control group) or day 3 of fifth-instar larvae 24 h after injection with 100 μg of dried *M*. *luteus* (induction group). The two groups of hemolymph were mixed with phenylthiourea (final concentration, 1 mM) to avoid melanization. Dried *M*. *luteus* was then added to the induction group hemolymph to a final concentration of 1 μg/μL to stimulate protease activation. After incubation for 30 min at room temperature with rocking, a protease inhibitor cocktail (Sigma P8849; 1 mL per 20 mL of hemolymph) was added to the two groups of samples. After incubation for 10 min at room temperature with rocking, the hemolymph mixtures were centrifuged at 5000 × g for 15 min at 4°C. The supernatants were then mixed with 200 μL of the cross-linked bead-Ab complexes and rotated horizontally at 4°C overnight. The hemolymph plus cross-linked bead-Ab mixture was applied to a Magnet to recover bead-Ab-target protein complexes. The complexes were then washed thrice times with 500 μL of PBST, and eluted with 100 μL of 50 mM glycine (pH 2.8) for 10 min at room temperature. The eluates were resolved by SDS-PAGE and analyzed by immunoblotting for target proteins or CBB-stained.

Based on the results of immunoblotting and CBB-staining, protein complex bonds were excised from the gel and identified as previously described [78]. Briefly, the bonds were digested with trypsin for 20 h at 37°C. The digested samples were lyophilized, resuspended in 0.1% formic acid, and analyzed using a Q Exactive Mass Spectrometer (Thermo Scientific). The resulting raw mass spectrometry (MS) data were analyzed using MaxQuant software [79]. The intensity-based absolute quantification (iBAQ) algorithm in MaxQuant was used to compare protein abundance.

### CRISPR/Cas9-mediated mutation, homozygote screening and molecular changes

To further verify the regulatory function of *serpin-1*, a unique single guide RNA (sgRNA) was designed for *serpin-1* and the CRISPR/Cas9 system was used to establishment *serpin-1* KO silkworm. CRISPR/Cas9 mediated *serpin-1* gene KO in silkworms was performed as described in our previous study [46]. Briefly, mixed *pBac*[3×P3-eGFP-U6-*serpin-1* s*gRNA*] (synchronously expressing green fluorescent protein and *serpin-1* sgRNA under the control of different promoters) and *piggyBac* helper plasmid (encoding piggyBac transposase) were microinjected into newly laid embryos of the N4 strain. Positive sgRNA transgenic embryos were screened for green fluorescent protein (GFP) using a fluorescence microscope (Nikon AZ100, Japan). The U6-*sgRNA* line targeting *serpin-1* was crossed with the nos-Cas9 transgenic line (stored in our laboratory) to generate F1 progeny and obtain chimeric mutants. Genotyping of *serpin-1* chimeric mutants was performed by amplification using gene-specific primers designed at the sides of the gRNA site. To obtain homozygous mutants of *serpin-1*, F1 moths (no fluorescence) with effectively edited forms were crossed with WT moths. Genomic DNA was extracted from the exuviae of each F2 individual for genotyping. Screened heterozygous individuals with the same effective editing genotype were crossed to obtain homozygotes. The screened KO homozygotes were designated as the *serpin-1* KO line. The hemolymph and fat bodies of WT and *serpin-1* KO individuals were collected on day 3 of the fifth-instar. Immunoblotting was used to detect the expression of serpin-1, serpin-6, and CLIP2 proteins in the hemolymph and fat bodies of WT and *serpin-1* KO individuals. The expression of AMP genes was detected by RT-qPCR in the fat bodies of WT and *serpin-1* KO individuals on day 3 of the fifth-instar and day 1 of the wandering stage.

Survival analysis was performed to determine whether knocking out *serpin-1* affects the survival capability of silkworm larvae following bacterial challenge. Thirty-five or 40 WT and *serpin-1* KO individuals on day 3 of the fifth-instar were randomly selected. The larvae were respectively injected with *E*. *coli*, *P*. *aeruginosa*, *M*. *luteus*, or *E*. *mundtii* (1×10^7^ CFU/mL, 10 μL) and larval survival was monitored every 12 h until 72 h after injection. The hemolymph of each group was collected 24 h post-injection to detect the expression of serpin-1, serpin-6, CLIP2 and gloverin2 proteins. To further analyze the effect of *serpin-1* on the immune homeostasis of silkworm, WT and *serpin-1* KO individuals were injected with 5 μL PBS (control group), LPS, or PGN. Eighteen hours post-injection, the hemolymph and fat bodies of each group were collected to detect the expression of serpins, CLIP2, and AMPs.

### Statistical analyses

The data processing of RT-qPCR and activity assays were performed using GraphPad Prism 6.0 (La Jolla, CA, USA). The Student’s *t*-test and one-way analysis of variance (ANOVA) were used to evaluate significant differences. Differences were considered significant at *P* < 0.05.

## Supporting information

S1 Data Excel spreadsheet containing the underlying numerical data for Figs 1–7 in separate sheets. (XLSX)

S1 Fig Specific determination of CLIP2 polypeptide antibody (A) and activation analysis of factor Xa on purified recombinant proCLIP2_Xa_ (B). (A) Immunoblot analysis of larval cell-free hemolymph and recombinant proCLIP2_Xa_ with CLIP2 polypeptide antibody. The black arrow indicates the CLIP2 protein, the red arrow indicates a covalent complex formed by CLIP2 and its specific inhibitor. (B) Purified recombinant proCLIP2_Xa_ (100 ng) and factor Xa (100–800 ng) were incubated for 20 min at room temperature and the mixtures were separated by SDS-PAGE, followed by immunoblot analysis using anti-His-tag antibodies. M: protein molecular weight marker. (TIF)

S2 Fig Domain analysis and prokaryotic expression refolding of silkworm proSpäetzle1 (proSpz1) protein. (A) Simple modular architecture research tool (SMART) used to predict the conserved domains and cleavage sites of proSpz1. (B) Recombinant proSpz1 protein in the precipitate was purified using a Ni-NTA column, and the purified recombinant proSpz1 inclusion body protein was renatured by dialysis against 20 mM PBS (pH 7.4) renaturation buffer with decreasing concentrations of urea. CL, crude extract; FT, flow-through; BB, binding buffer; 50–1000: elution fractions of the stepwise imidazole gradient. Arrow indicates the recombinant proSpz1 protein. M: protein molecular weight marker.
(TIF)

S3 Fig Purification and inhibitory activity assay for serpin-1a and serpin-6. (A and B) Purification and inhibitory activity assays of serpin-1a. (C, D) Purification and inhibitory activity assays for serpin-6. M, Marker; CL, Crude protein liquid; FT, flow-through; BB, elution fraction of binding buffer; 20–1000, elution fraction of stepwise imidazole gradient. M: protein molecular weight marker. (E) Inhibitory activity analysis of serpin-1a and its mutants (E329A, R340A and S341A). (F) SDS-stable complex formation between CLIP2 and serpin-1a mutants. CLIP2 (200 ng) was incubated with corresponding serpins at room temperature for 5 min under a molar mass ratio of 1:5 (CLIP2: serpins). The samples were subjected to SDS-PAGE and immunoblot analysis using antibodies against CLIP2. The black arrow indicates the rCLIP2, the red arrow indicates the CLIP2-serpin complex.
(TIF)

S4 Fig Effects of pathogen-associated molecular patterns (PAMPs) on *serpin* expression in silkworm fat body. For PAMP induction, the larvae of each group were injected with 5 μL of PBS (CK), 2.5 μg of lipopolysaccharide (LPS), peptidoglycan (PGN), or curdlan (CDN). Twelve hours post-injection, the fat bodies of the silkworms were collected for RNA isolation. Transcript levels of *Spz1* (upper), antibacterial peptide genes (upper), and serpins (lower) were detected using RT-qPCR. Error bars represent mean ± SD (*n* = 3). *P < 0.05, **P < 0.01, ***P < 0.001.
(TIF)

S5 Fig CRISPR/Cas9-mediated *serpin-1* knockout. (A) Schematic diagram of serpin-1 gRNA location. Screening strategy for *serpin-1* knockout (*serpin-1*^*-/-*^) homozygotes (B) and genomic DNA sequencing (C).
(TIF)

S6 Fig Immunoregulatory function analysis of recombinant serpin-1a protein in silkworm. (A) The recombinant serpin-1a protein with different masses was incubated with 5 μL of plasma from day-3 fifth instar larvae at room temperature for 30 min, and spontaneous melanization was recorded. (B) Screened hemolymph from *B*. *mori* larvae was first mixed with bovine serum albumin (BSA), serpin-1a, and phenylthiourea (PTU); *Micrococcus luteus* was then added and incubated at room temperature for 20 min, after which the PO activity of each group was determined using L-dopa as a substrate. Error bars represent mean ± SD (*n* = 3). Different letters represent significant differences (one-way ANOVA followed by Tukey’s test; *P* < 0.05). (C) Serpin-1a inhibits *Micrococcus luteus*-induced expression of antimicrobial peptides in silkworm fat bodies. Day-3 fifth instar larvae were injected with serpin-1a (5 μL, 3 μg/μL) or BSA (15 μL, 1 μg/μL). After 30 min, larvae were administered a second injection with *M*. *luteus* (5 μL, 0.5 μg/μL), and the fat bodies of each group were collected 2 h after the second injection. The transcript levels of the antibacterial peptide genes were determined using RT-qPCR. (D) Effect of starvation on the expression of *serpin-1a* in the fat body. To test the influence of starvation on the expression of *serpin-1a*, newly molted fifth instar larvae were divided into two groups. Feeding group: larvae were collected at 12, 24, 36, and 48 h after being fed mulberry leaves. Starvation group: larvae were collected at 12, 24, 36, and 48 h post-starvation without mulberry leaves. The larval fat body in each group were collected for analysis. Error bars represent mean ± SD (*n* = 3). *P < 0.05, **P < 0.01, ***P < 0.001.
(TIF)

S1 Table Proteins and their abundances identified by LC-MS/MS from the immunoprecipitated purified CLIP2-inhibitor complexes. (XLSX)

S2 Table The primers used in this study. (XLSX)
