# Supplementary figures and images for: Serpin-1a and serpin-6 regulate the Toll pathway immune homeostasis by synergistically inhibiting the Spätzle-processing enzyme CLIP2 in silkworm, Bombyx mori
Source: PLoS Pathog. 2023 Oct 18;19(10):e1011740. doi: 10.1371/journal.ppat.1011740 (PMC10629668; doi:10.1371/journal.ppat.1011740)

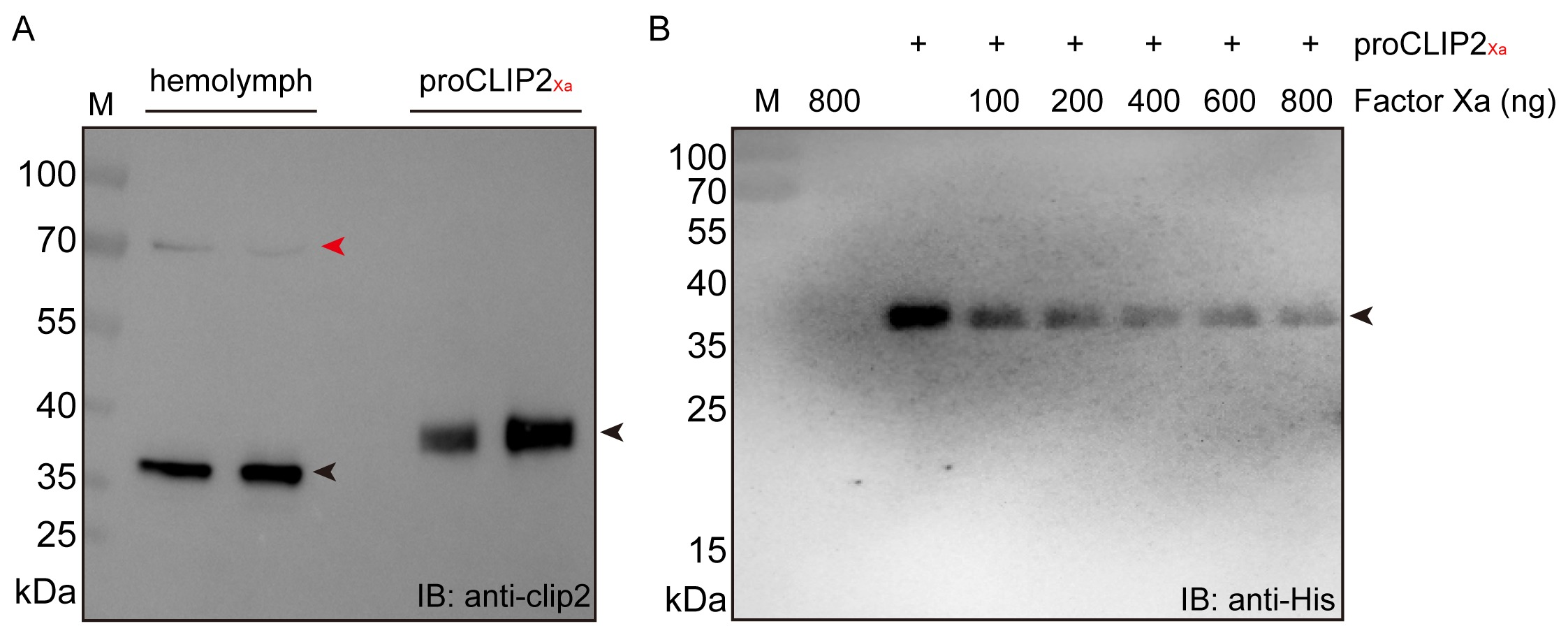

Supplement: S1 Fig — (TIF) [file ppat.1011740.s002.tif]

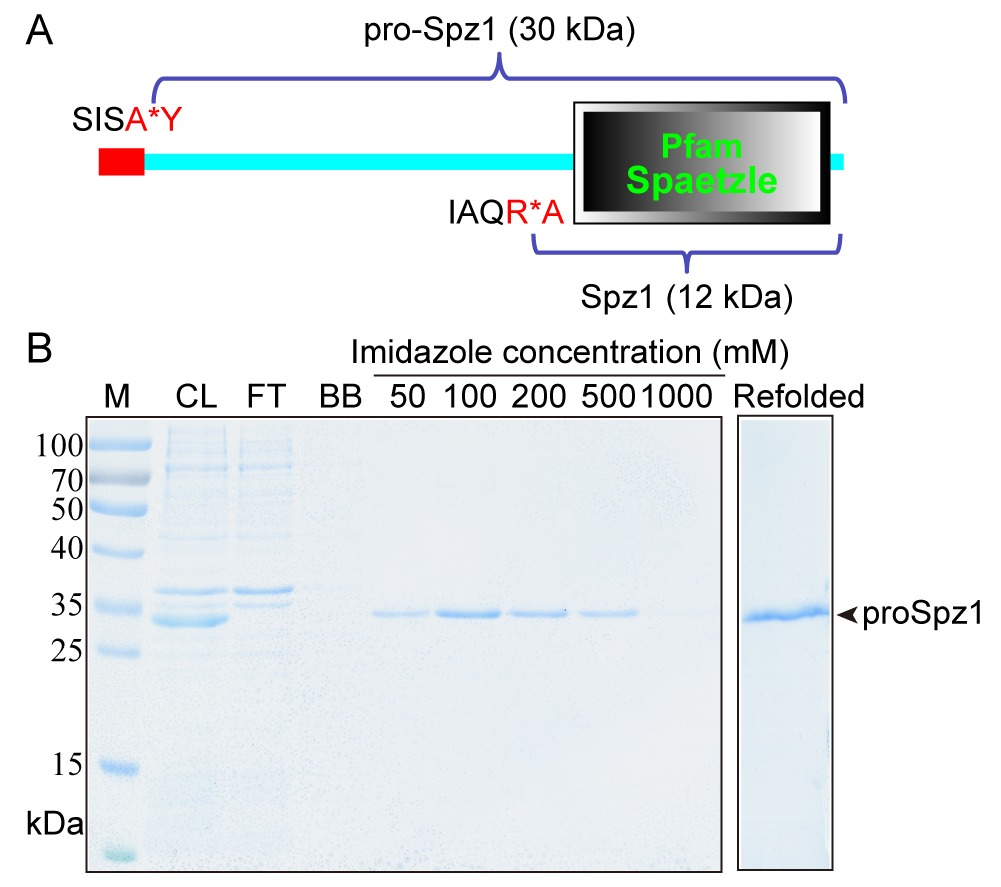

Supplement: S2 Fig — (A) Simple modular architecture research tool (SMART) used to predict the conserved domains and cleavage sites of proSpz1. (B) Recombinant proSpz1 protein in the precipitate was purified using a Ni-NTA column, and the purified recombinant proSpz1 inclusion body protein was renatured by dialysis against 20 mM PBS (pH 7.4) renaturation buffer with decreasing concentrations of urea. CL, crude extract; FT, flow-through; BB, binding buffer; 50–1000: elution fractions of the stepwise imidazole gradient. Arrow indicates the recombinant proSpz1 protein. M: protein molecular weight marker. (TIF) [file ppat.1011740.s003.tif]

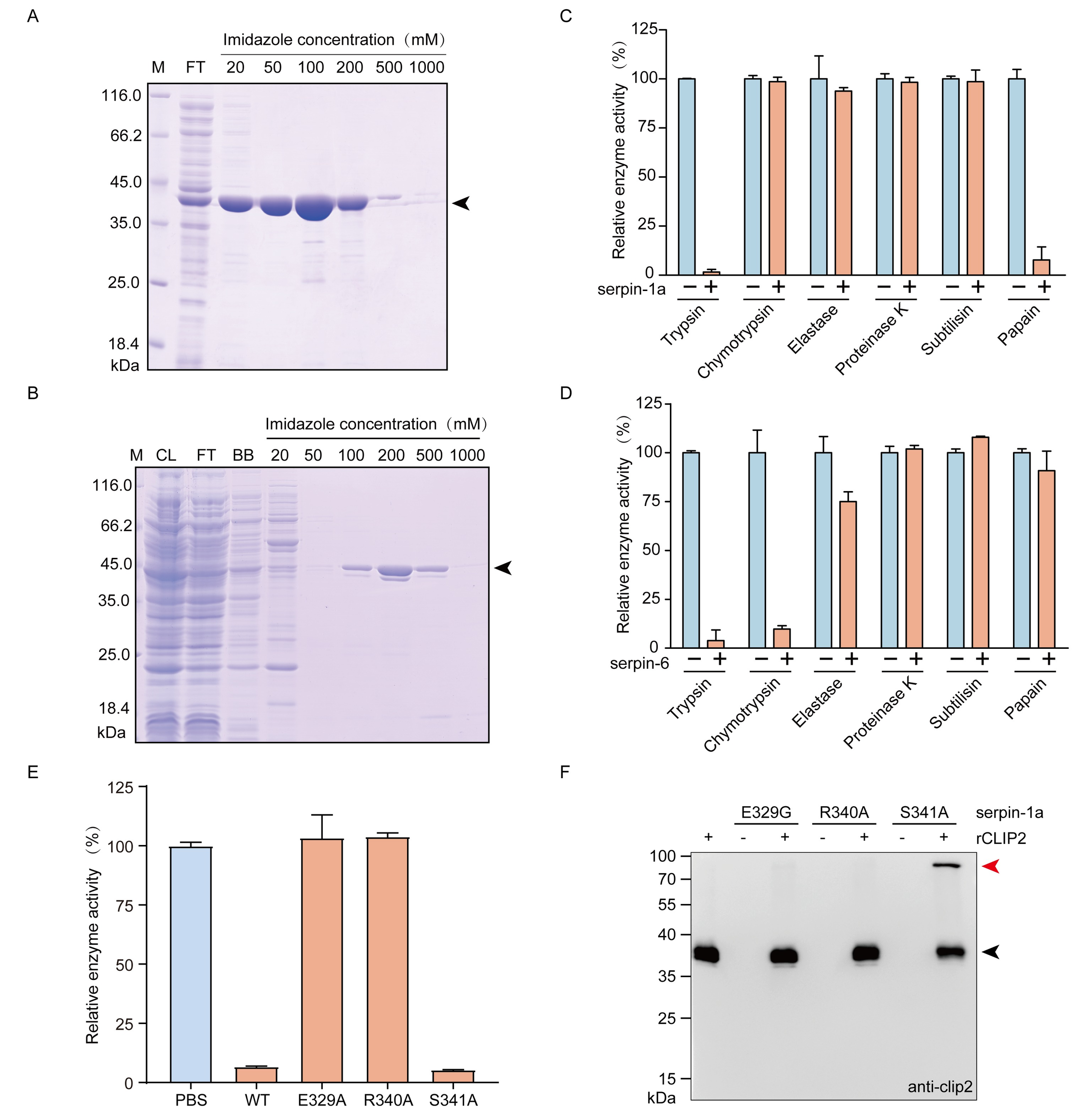

Supplement: S3 Fig — (A and B) Purification and inhibitory activity assays of serpin-1a. (C, D) Purification and inhibitory activity assays for serpin-6. M, Marker; CL, Crude protein liquid; FT, flow-through; BB, elution fraction of binding buffer; 20–1000, elution fraction of stepwise imidazole gradient. M: protein molecular weight marker. (E) Inhibitory activity analysis of serpin-1a and its mutants (E329A, R340A and S341A). (F) SDS-stable complex formation between CLIP2 and serpin-1a mutants. CLIP2 (200 ng) was incubated with corresponding serpins at room temperature for 5 min under a molar mass ratio of 1:5 (CLIP2: serpins). The samples were subjected to SDS-PAGE and immunoblot analysis using antibodies against CLIP2. The black arrow indicates the rCLIP2, the red arrow indicates the CLIP2-serpin complex. (TIF) [file ppat.1011740.s004.tif]

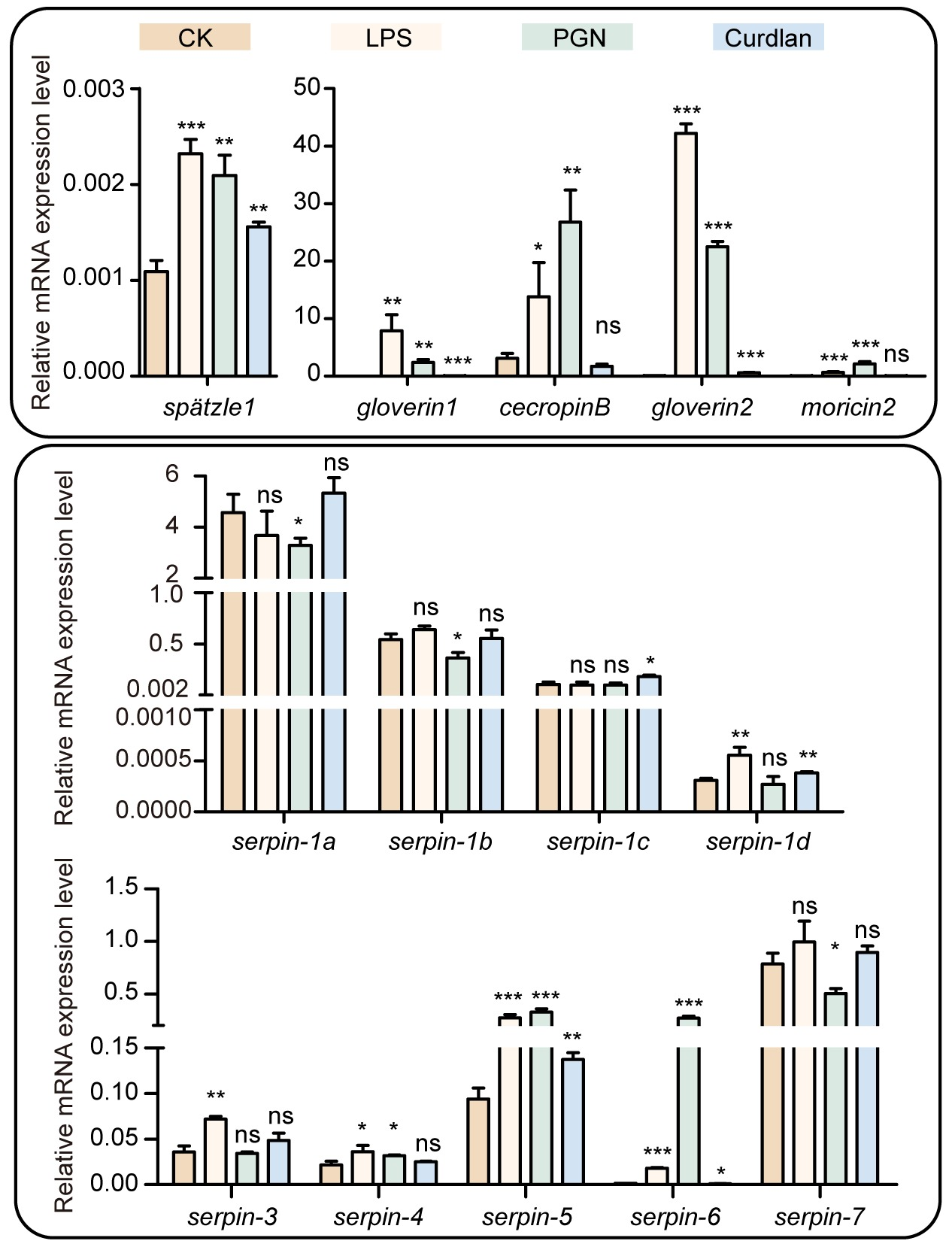

Supplement: S4 Fig — For PAMP induction, the larvae of each group were injected with 5 μL of PBS (CK), 2.5 μg of lipopolysaccharide (LPS), peptidoglycan (PGN), or curdlan (CDN). Twelve hours post-injection, the fat bodies of the silkworms were collected for RNA isolation. Transcript levels of Spz1 (upper), antibacterial peptide genes (upper), and serpins (lower) were detected using RT-qPCR. Error bars represent mean ± SD (n = 3). *P < 0.05, **P < 0.01, ***P < 0.001. (TIF) [file ppat.1011740.s005.tif]

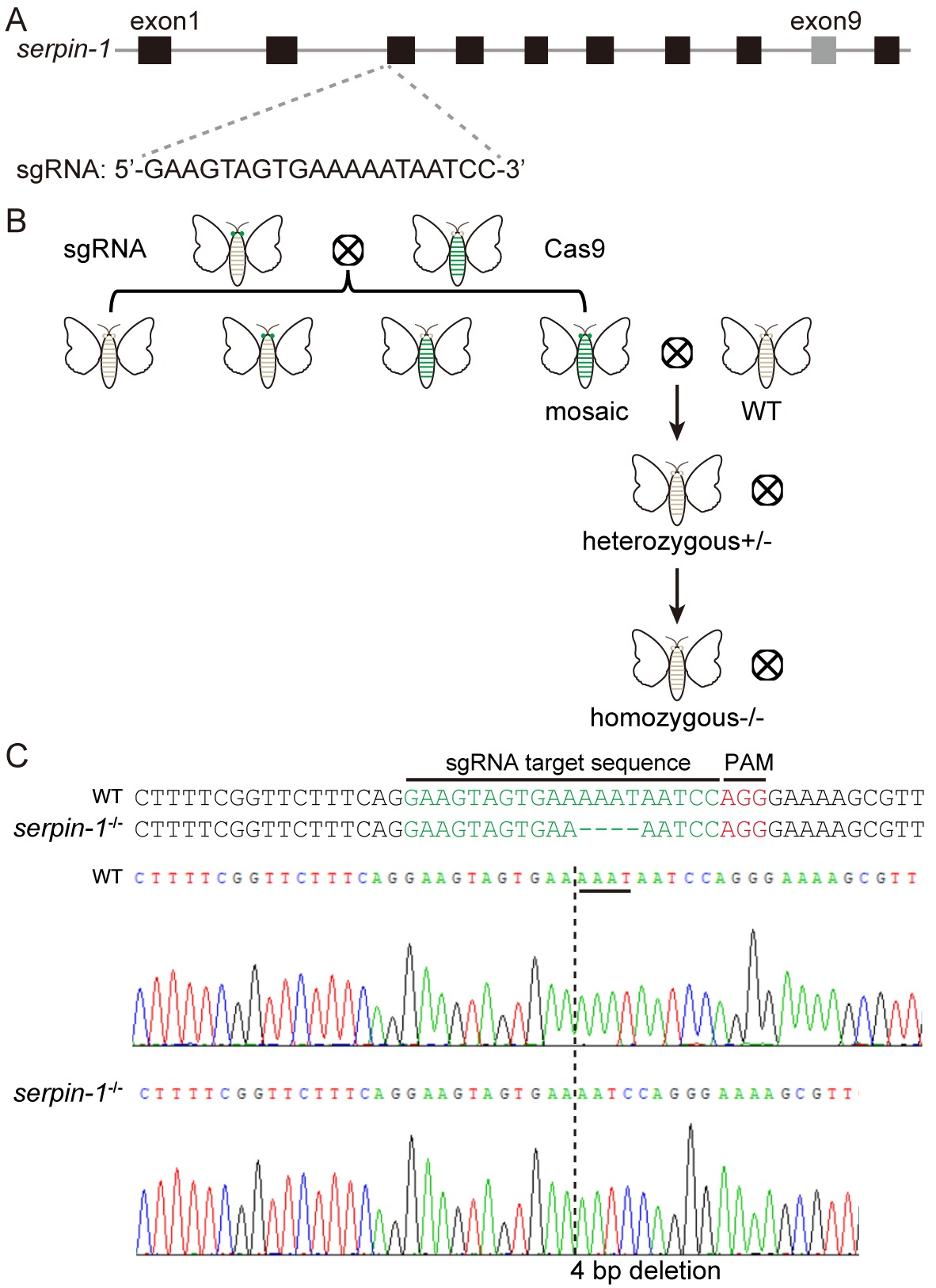

Supplement: S5 Fig — (A) Schematic diagram of serpin-1 gRNA location. Screening strategy for serpin-1 knockout (serpin-1-/-) homozygotes (B) and genomic DNA sequencing (C). (TIF) [file ppat.1011740.s006.tif]

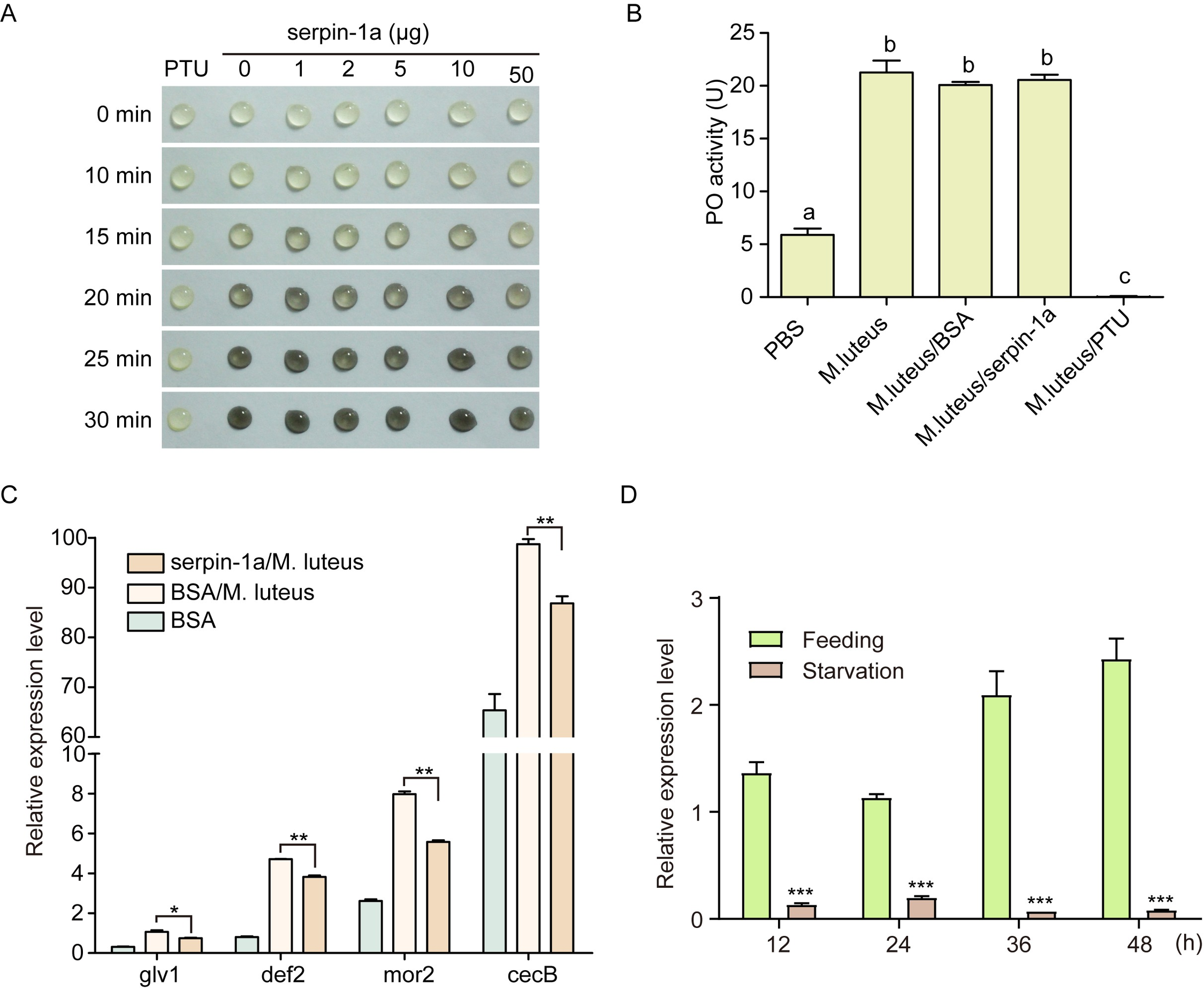

Supplement: S6 Fig — (A) The recombinant serpin-1a protein with different masses was incubated with 5 μL of plasma from day-3 fifth instar larvae at room temperature for 30 min, and spontaneous melanization was recorded. (B) Screened hemolymph from B. mori larvae was first mixed with bovine serum albumin (BSA), serpin-1a, and phenylthiourea (PTU); Micrococcus luteus was then added and incubated at room temperature for 20 min, after which the PO activity of each group was determined using L-dopa as a substrate. Error bars represent mean ± SD (n = 3). Different letters represent significant differences (one-way ANOVA followed by Tukey’s test; P < 0.05). (C) Serpin-1a inhibits Micrococcus luteus-induced expression of antimicrobial peptides in silkworm fat bodies. Day-3 fifth instar larvae were injected with serpin-1a (5 μL, 3 μg/μL) or BSA (15 μL, 1 μg/μL). After 30 min, larvae were administered a second injection with M. luteus (5 μL, 0.5 μg/μL), and the fat bodies of each group were collected 2 h after the second injection. The transcript levels of the antibacterial peptide genes were determined using RT-qPCR. (D) Effect of starvation on the expression of serpin-1a in the fat body. To test the influence of starvation on the expression of serpin-1a, newly molted fifth instar larvae were divided into two groups. Feeding group: larvae were collected at 12, 24, 36, and 48 h after being fed mulberry leaves. Starvation group: larvae were collected at 12, 24, 36, and 48 h post-starvation without mulberry leaves. The larval fat body in each group were collected for analysis. Error bars represent mean ± SD (n = 3). *P < 0.05, **P < 0.01, ***P < 0.001. (TIF) [file ppat.1011740.s007.tif]
